# Endothelial ADAM17 facilitates neutrophil migration and pulmonary microvascular permeability in acute lung inflammation

**DOI:** 10.1186/s12929-026-01290-7

**Published:** 2026-09-28

**Authors:** Anna Biedritzky, Carolin Kleinmaier, Anika Fuhr, Lisa Maria Herrmann, Kristian-Christos Ngamsri, Franziska Konrad, Michael Koeppen

**Affiliations:** 1 Department of Anesthesiology and Intensive Care Medicine, University Hospital of Tuebingen, University of Tuebingen, Hoppe-Seyler-Strasse 3, 72076 Tuebingen, Germany; 2 Center for Anesthesiology, Intensive Care Medicine, Perioperative Medicine and Pain Therapy Markgröningen, Klinik für Anästhesiologie, Orthopädische Klinik Markgröningen, RKH Regional Clinics Holding and Services GmbH, Kurt-Lindemann-Weg 10, 71706 Markgröningen, Germany

**Keywords:** Acute respiratory distress syndrome, Endothelial ADAM17, Microvascular permeability, Endothelial barrier dysfunction, Neutrophil migration, Pulmonary inflammation

## Abstract

**Background:**

Acute respiratory distress syndrome (ARDS) is characterized by endothelial barrier disruption, excessive neutrophil recruitment, and sustained pulmonary inflammation. The A Disintegrin and Metalloproteinase 17 (ADAM17) regulates inflammatory signaling through ectodomain shedding of adhesion molecules and cytokine receptors, yet its endothelial-specific contribution to acute lung injury remains poorly defined.

**Methods:**

Endothelial ADAM17 was deleted in ADAM17^fl/fl^ Tie2-Cre^+^ mice using the Cre/loxP system. Acute lung inflammation was induced by lipopolysaccharide (LPS) inhalation. Systemic ADAM17 blockade was achieved by intraperitoneal TAPI‑1 or ADAM17/ADAM10 inhibition by GW280264X, administered 1 h before LPS. Pulmonary microvascular permeability was quantified by Evans Blue extravasation. Neutrophil trafficking across lung compartments (intravascular, endothelial adherent, interstitial, intra‑alveolar) was assessed by flow cytometry.

**Results:**

Endothelial ADAM17 emerged as a central regulator of vascular permeability, neutrophil trafficking, and inflammatory amplification in LPS-induced acute pulmonary inflammation. LPS markedly increased pulmonary ADAM17 expression, whereas endothelial-specific deletion reduced total lung ADAM17 mRNA by 77.5%, highlighting the dominant endothelial contribution. Endothelial ADAM17 deficiency preserved endothelial junction integrity through maintenance of JAM-A surface expression and was associated with preserved expression of VE-cadherin and the tight junction proteins ZO-1, occludin, and claudin-5, thereby reducing protein-rich pulmonary edema. These changes were accompanied by enhanced neutrophil transmigration into interstitial and alveolar compartments through altered PSGL-1 and CD49d expression. Mechanistically, endothelial ADAM17 enhanced TNF receptor 1 and IL-6 receptor signaling, elevating TNF-α, myeloperoxidase, and neutrophil elastase levels. Pharmacological ADAM17 inhibition recapitulated the protective phenotype of endothelial ADAM17 deficiency, attenuating neutrophil recruitment, preserving endothelial barrier integrity, and limiting vascular leakage.

**Conclusion:**

These findings strengthen the evidence that endothelial ADAM17 is a key driver of inflammatory vascular dysfunction in ARDS and support ADAM17 as a rational therapeutic target.

**Supplementary Information:**

The online version contains supplementary material available at https://doi.org/10.1186/s12929-026-01290-7.

## Background

Acute respiratory distress syndrome (ARDS) is a rapidly progressive and often fatal condition characterized by non-cardiogenic pulmonary edema, profound hypoxemia, and widespread alveolar inflammation. Despite advances in supportive care, mortality in severe ARDS remains high, reaching up to 40% [1]. At the core of ARDS pathophysiology is a sustained hyperinflammatory response that disrupts the alveolar–capillary barrier, increases microvascular permeability, and drives neutrophil infiltration into the lung parenchyma.

Neutrophils and endothelial cells engage in dynamic interactions during acute inflammation. While neutrophils mediate pathogen clearance, their excessive accumulation contributes to tissue damage and worsens pulmonary edema. However, the molecular mechanisms governing neutrophil recruitment and endothelial barrier dysfunction in ARDS remain incompletely understood.

A Disintegrin and Metalloproteinase 17 (ADAM17) is a membrane-bound protease that modulates inflammation through the ectodomain shedding of adhesion molecules and cytokine receptors, including TNF-α, L-selectin, and junctional adhesion molecule A (JAM-A). Prior studies have implicated ADAM17 in leukocyte trafficking and barrier regulation across various inflammatory settings, and its pharmacological inhibition has shown promise in preclinical models of lung injury. Notably, Dreymueller et al. demonstrated that endothelial-specific ADAM17 deficiency attenuates LPS-induced pulmonary vascular permeability, leukocyte recruitment, JAM-A shedding, and inflammatory cytokine release, establishing endothelial ADAM17 as an important regulator of acute lung inflammation [12]. However, the context-dependent functions of ADAM17 limit the therapeutic potential of global inhibition, and several mechanistic aspects of endothelial ADAM17 signaling remain incompletely understood. Since endothelial cells form the first interface for leukocyte transmigration and actively contribute to inflammatory signaling, dissecting the role of ADAM17 in this compartment is critical to understanding the pathogenesis of ARDS and identifying viable therapeutic targets.

Here, we tested the hypothesis that endothelial ADAM17 promotes neutrophil recruitment, disrupts endothelial barrier integrity, and amplifies pulmonary inflammation in ARDS. Using a conditional, endothelial-specific ADAM17 knockout mouse model and two pharmacological inhibitors (TAPI-1 and GW280264X), we systematically investigated how endothelial ADAM17 modulates microvascular permeability, neutrophil trafficking across distinct pulmonary compartments, inflammatory signaling pathways, and cytokine release in a murine model of LPS-induced acute lung injury.

## Methods

### Animals

The Adam17^tm1.2Bbl^/J floxed mice (strain: 009597; RRID:IMSR_JAX:009597, MGI ID: MGI:4,354,847) were obtained from Jackson Laboratory (Bar Harbor, USA). This mouse line contains loxP sites flanking exon 2 of the ADAM17 locus. To generate the tissue-specific knockout, these mice were crossed with Tie2-Cre mice (Tg(Tek-cre)12Flv; MGI ID: MGI: 2,136,412) kindly provided by Prof. Dr. Peter Rosenberger, resulting in the B6.129P2-ADAM17tm1.2Bbl-Tek-cre1Ywa/J also referred to as ADAM17^fl/fl^ Tie2-Cre^+^. The crossing of these lines produced a tissue-specific ADAM17 knockout in murine endothelial cells via Cre-mediated deletion of the floxed ADAM17 gene. The Cre-negative littermates were used as control animals and will be referred to as ADAM17^fl/fl^ Tie2-Cre^−^. Female and male animals were used in a 1:1 ratio at eight to twelve weeks age. All animal experiments were conducted in accordance with the ARRIVE guidelines (ARRIVE 2.0) and the European Directive 2010/63/EU for the protection of animals used for scientific purposes. All animal protocols were approved by the Regierungspräsidium Tübingen (A05/21G) under the German Animal Welfare Act and adhered to the principles of the 3Rs (Replacement, Reduction, Refinement) to ensure ethical standards.

### Murine model of LPS-induced acute pulmonary inflammation and drug administration

Acute pulmonary inflammation was induced via LPS inhalation (0,5 mg/ml in 7 ml sterile saline, derived from *Salmonella enterica* serotype enteritidis, Sigma Aldrich) for 30 min in a custom-built chamber. Nebulized LPS resulted in a transient pulmonary inflammation characterized by increased neutrophil migration, elevated microvascular permeability and altered chemokine release. For systemic ADAM17 inhibition in ADAM17^fl/fl^ Tie2-Cre^−^, TAPI-1 (5 µg/g body weight) and GW280264X (30 µg/g body weight) were administered intraperitoneally one hour before LPS inhalation. Identical ADAM17^fl/fl^ Tie2-Cre^−^ control animals were used for comparison to both endothelial ADAM17 deletion and systemic inhibition, in accordance with the 3Rs principle from Russell and Burch (1959) to minimize animal numbers. For transparency, this is noted in the relevant figure legend.

### Immunofluorescence

Paraffin-embedded lung sections were deparaffinized and rehydrated. Lung sections and cultured HMEC-1 (CRL-3243™, ATCC) and H441 (NCBI-H441, CRM-HTB-174™, ATCC) cells were fixed in 4% PFA. After washing, both tissue sections and cultured cells were permeabilized with 0.1% Triton X-100 and blocked with 5% bovine serum albumin (BSA) in PBS to reduce nonspecific binding. Sections and cells were then incubated with primary antibodies targeting ADAM17 (rabbit anti-ADAM17, polyclonal, Bioss, USA), Ly6G/C (rat anti-Ly6G/C, clone RB6-8C5, Abcam, UK), VE-cadherin (goat anti-VE-cadherin, polyclonal, R&D Systems, USA), ZO-1 (rabbit anti-ZO-1, polyclonal, Invitrogen, USA), occludin (mouse anti-Occludin, clone OC-3F10, Invitrogen, USA), claudin-5 (rabbit anti-claudin-5, polyclonal, Invitrogen, USA), TLR4 (rabbit anti-TLR4, polyclonal, Invitrogen, USA), TNFR1 (mouse anti-TNFR1, clone H-5, Santa Cruz, USA), IL-6Rα (mouse anti-IL-6Rα, clone H-7, Santa Cruz, USA), vWF (sheep anti-vWF, polyclonal, Abcam, UK) and F-actin (Phalloidin Labeling, Invitrogen, USA) followed by incubation with the corresponding fluorescently labelled secondary antibodies including Alexa Fluor 488 goat anti-rabbit, Alexa Fluor 647 goat anti-rat, Alexa Fluor 488 donkey anti-goat, Alexa Fluor 594 goat anti-mouse, Alexa Fluor 488 donkey anti-mouse and Alexa Fluor 647 donkey anti-sheep. Nuclear counterstaining was performed using Roti-Mount FluorCare containing DAPI (4´,6-diamidino-2-phenylindole; HP20.1, Carl Roth, Germany). Images were acquired using a Leica Stellaris 8 confocal microscope and processed with LasX software. For each experimental condition, four independent experiments were performed, each including two technical replicates. Images were analyzed using ImageJ (National Institues of Health, USA, 1.54d).

### *Flow cytometry-based *in vivo* migration assay*

Neutrophil migration into the vascular, endothelial adherent, interstitial, and bronchoalveolar compartments of the inflamed lung was determined by flow cytometry 24 h after LPS inhalation. For the compartment-specific migration analysis, blood, lung tissue, and bronchoalveolar lavage (BAL) fluid were collected to quantify intravascular, endothelial-adherent, interstitial, and bronchoalveolar neutrophils, respectively. PMNs attached to the pulmonary endothelium were tagged by an allophycocyanin (APC)-conjugated anti-Ly6G antibody (clone 1A8, BioLegend, USA), which was injected intravenously into the tail vein immediately prior to anaesthesia. This previously established and validated approach labels neutrophils within the pulmonary vasculature, including endothelial-adherent PMNs, while minimizing labeling of transmigrated interstitial neutrophils [2–10]. In deep anaesthesia, thoracotomy was performed and followed by blood collection and extensive perfusion of the pulmonary vasculature with PBS to remove circulating PMNs. Alveolar PMNs were collected via bronchoalveolar lavage. Lung tissue was collected, enzymatically digested using collagenase, DNAse and hyaluronidase (Sigma-Aldrich, Germany) and subsequently homogenized. Bronchoalveolar lavage (BAL) was centrifuged and cells were resuspended in staining buffer. The whole blood and cell suspensions of lung and BAL were stained with PerCP-conjugated CD45 (clone 30-F11, BioLegend) and PE/Cy7-conjugated Ly6G (clone 1A8, BioLegend) antibodies. Neutrophils in blood and BAL samples were identified as PerCP-CD45⁺/PE-Cy7-Ly6G⁺ cells. Within lung tissue, endothelial-adherent PMNs were identified as intravenously labeled PerCP-CD45⁺/PE-Cy7-Ly6G⁺/APC-Ly6G⁺ cells, whereas interstitial PMNs were defined as PerCP-CD45⁺/PE-Cy7-Ly6G⁺/APC-Ly6G⁻ cells (Supplementary Fig. 1). Additionally, the surface expression of the adhesion molecules CD162 (PSGL-1), CD49d and CD321 (JAM-A) as well as ADAM17 was assessed using the following antibodies: PE-conjugated CD162 (clone 2PH1, BioLegend), APC-conjugated CD49d (clone R1-2, BioLegend), PE-conjugated CD321 (clone 27-9, BioLegend), and PE-conjugated ADAM17 (polyclonal, BioLegend).

### Bicinchoninic acid assay for total protein determination

The total protein amount in BAL supernatants was measured as an indirect indicator of microvascular leakage. For this purpose, the BCA assay kit (Pierce Thermo Scientific, USA) was used according to the manufacturer´s instructions.

### Evans blue extravasation assay

For the determination of endothelial barrier dysfunction and microvascular leakage, Evans blue was injected intravenously into the tail vein at a dose of 20 µg/g body weight, five hours and 30 min after LPS-inhalation. After 30 min incubation, mice were anesthetized, blood collected and subsequently perfused with PBS to remove residual blood from the pulmonary circulation. Lungs were excised, weighed, and diluted with PBS. Tissue was homogenized using ceramic beads and homogenates were mixed 1:2 with formamide and incubated at 60 °C for 12–16 h. Blood samples were centrifuged, plasma was collected and stored at 4 °C overnight. At the following day, lung samples were centrifuged and optical density was determined at 620 nm and 740 nm as reference wavelength. Evans blue concentrations in plasma and lung were determined by regression analysis using the standard curve.

### Immunohistochemistry

To evaluate neutrophil accumulation in the lung 24 h after LPS exposure, mice were anesthetized and subsequently perfused to remove residual blood from the pulmonary circulation. Lungs were inflated and fixed via tracheal cannulation with 4% formaldehyde for 10 min at a pressure of 25 cm H_2_O, excised, embedded in paraffin, and sectioned at thickness of 3 µm. For immunohistochemical analysis, sections were first blocked with Avidin solution (Vector Laboratories, USA) and then incubated overnight at 4 °C with a rat anti-mouse Ly6G/C antibody (1:500, clone RB6-8C5, Abcam, UK). After washing, sections were incubated for 1 h with a biotinylated anti-rabbit IgG secondary antibody (BA400, Vector Laboratories), followed by visualization with 3,3′-diaminobenzidine (DAB) substrate (SK-4100, Vector Laboratories). Nuclear counterstaining was performed with Mayer´s hematoxylin (1.09249, Sigma Aldrich, Germany). Images were acquired with a Leitz DM IRB microscope (Leica) and processed using AxioVision software (v4.8.2), while quantitative image analysis was carried out with ImageJ (v1.53 k).

### Gene expression

Total RNA was extracted from murine lung tissue three hours following LPS inhalation using TRIzol reagent (Invitrogen, Carlsbad, USA) in accordance with the manufacturer´s protocol. Subsequently, complementary DNA (cDNA) was synthesized from the isolated RNA employing the iScript cDNA synthesis kit (Bio-Rad, Munich, Germany) following the manufacturer´s instructions. Quantitative real-time PCR was then performed to determine relative gene expression levels using the following primers: ADAM17 (5ʹ-gag agc cat cag ttt g-3ʹ, 5ʹ-ccc tcc atg agt ttg c-3ʹ), VE-cadherin (5'-gtg ctc tcc aca aag ctc gg-3', 5'-gga gga gct gat ctt gtc cg-3'), iRhom2 (5'-gac acc ttc gac tcc tcc tt-3', 5'-ccg ctg tat tct ttt agc ggg − 3'), AT1R ( 5'-tgc cgt gaa ctt gaa ggt tat tc-3', 5'-gct tga gaa cac caa gca gc-3'), iTAP (5'-tcc aga act gga ggc ctg a-3', 5'-ctt ctg ccc cct cca tct tg-3'), TNFR1 (5'-ctt cag cac ccc agg ctt ta-3', 5'-tcg caa ggt ctg cat tgt ca-3'), TNFR2 (5'-cta agt gtc ctc ctg gcc aat − 3', 5'-tgg gtt ttc aag gcg cag ta-3'), TRAF2 (5'-gct act gct cct tct gcc tg-3', 5'-gca ggt tct cag tct cca cc-3'), MAPK1 (5'-tga agt tga aca ggc tct ggc − 3', 5'-tcc tct gag ccc ttg tcc tga − 3'), MAPK3 (5'-gta cgg cat ggt cag ctc ag-3', 5'-ctg agg atg tct cgg atg cc-3'), Nf-κB (5'-gcc aag tgc acg agt c-3', 5'-gaa gga cga gac gag c-3'), p38 (5'-agc tgg tag cgg tgc gat aa-3', 5'-cat ctc tct ctg tcc agt gcc a-3'), JNK (5'-agc cgt ctc ctt tag cac ag-3', 5'-tgt atc cga ggc caa agt cg-3'), FADD (5'-ctc aat cgc ctt ccg cat tg-3', 5'-aca gcc agg tga gaa atg gg-3'), TRADD (5'-tct gca ggt tcg aag ttc cc-3', 5'-gtg gcc ggt tca cta cga g-3'), CREB (5'-gag cag aca acc agc aga gt-3', 5'-tgg cat gga tac ctg ggc ta-3'), IL-6R (5'-tca ctg tgc gtt gca aac ag-3', 5'-gat ccg gct gca cca ttt tt-3'), JAK1 (5'-gcc tgt cta ctc cat gag cc-3', 5'-gtc tgg atc ttg cct ggt ca-3'), STAT3 (5'-tca gcg aga gca gca aag aa-3', 5'-ctt ggt ctt cag gta cgg gg-3'), TRIF (5'-ctt ccc cac agt ccc aat cc-3', 5'-gaa cca tct ggg cat ggt ga-3'), TRAM (5'-tct atg ctt gtc ctc gca cc-3', 5'-gga gcg act act ttc cca gg-3').

### ELISA

Levels of the inflammatory mediators TNF-α, CD62L, IL-10, ELA2, and CXCL2/3 were quantified in BAL three hours after LPS inhalation. ELISAs were performed with kits from R&D Systems (USA) according to the manufacturer´s protocol. Levels of soluble VE-cadherin were evaluated using the kit from Abcam (UK).

### MPO

The release of myeloperoxidase, serving as an indicator of neutrophil activity, was quantified in the supernatant of BAL at 24 h following LPS inhalation. Quantification of MPO activity was performed using a colorimetric assay, based on the peroxidase-mediated conversion of 2,2ʹ-Azino-di(3-ethylbenzthiazolin-6-sulfonsäure) (ABTS), with optical density determined at 405 nm using a microplate reader (Infinite M200 PRO, Tecan, Switzerland).

### In vitro* neutrophil transmigration assay*

To complement the in vivo compartment-specific migration analysis, an in vitro transendothelial–transepithelial neutrophil transmigration assay was performed as previously described [10]. Human epithelial cells H441 (NCBI-H441, CRM-HTB-174™, ATCC) were cultured on the bottom side and HMEC-1 endothelial cells (CRL-3243™, ATCC) on the upper side of Transwell inserts (Corning, USA). Freshly isolated human neutrophils (ethics approval no. 483/2021BO2) were added to the upper chamber and allowed to migrate across both cellular monolayers along a chemotactic gradient generated by N-formylmethionine-leucyl-phenylalanine (fMLP, 10 ng/mL; Sigma-Aldrich, Germany). Endothelial and epithelial monolayers were pretreated for 1 h with vehicle, the ADAM17-preferring inhibitor TAPI-1 or the combined ADAM10/17 inhibitor GW280264X prior to stimulation. After 1 h, transmigrated neutrophils were collected and analyzed by flow cytometry for the surface expression of CD11b and JAM-A. Myeloperoxidase (MPO) release into the supernatant was determined by MPO assay. The inserts containing the remaining adherent cells were fixed in 4% formalin, embedded in paraffin, sectioned, and processed for immunohistochemical staining.

### Statistical analysis

Statistical analyses were performed using GraphPad Prism software (v10.1, GraphPad Software, San Diego, USA). Unless stated otherwise, data are presented as mean ± standard deviation (SD). Outlier detection was conducted using the Robust Regression and Outlier Removal (ROUT) method with a coefficient of Q = 1%. The normality of data distribution was evaluated using both the D´Agostino-Pearson test and the Shapiro–Wilk test. For normally distributed data, group comparisons were carried out using one-way analysis of variance (ANOVA), followed by Bonferroni´s post hoc test. In cases where data did not meet normality assumptions, the non-parametric Kruskal–Wallis test was applied. All statistical tests were two-tailed, and values of p < 0.05 were considered statistically significant.

## Results

### ADAM17 expression is upregulated during LPS-induced acute pulmonary Inflammation

**Fig. 1:**
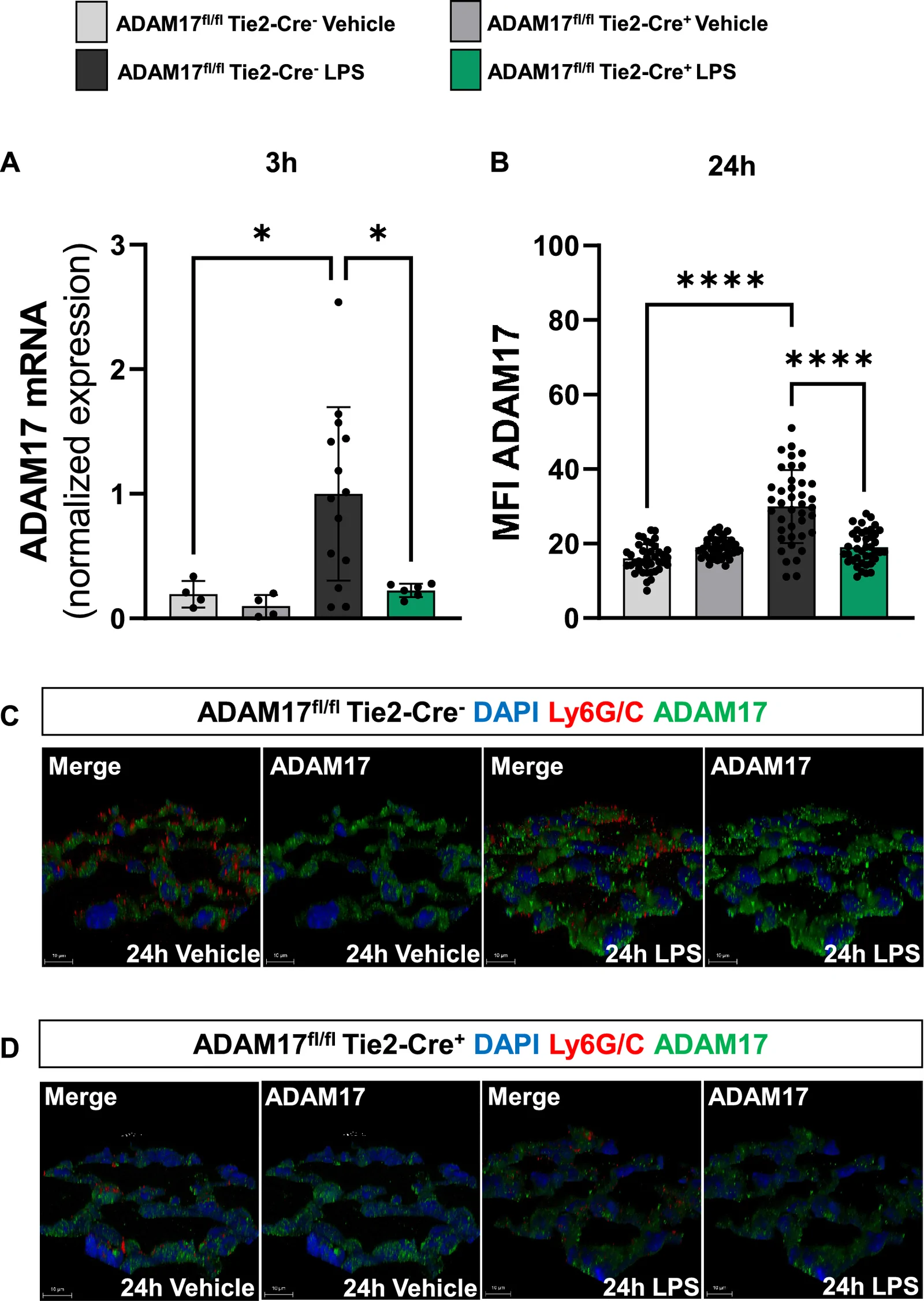
ADAM17 expression is upregulated during LPS-induced acute pulmonary inflammation. **A** Gene expression of ADAM17 in lung tissue three hours after LPS inhalation, measured by RT-qPCR (n = 4/4/14/8 mice per group). Expression data were normalized to the ADAM17^fl/fl^ Tie2-Cre^−^ LPS control group, whose mean value was set to 1. **B** Quantification of ADAM17 mean fluorescence intensity (MFI) in lung tissue using ImageJ software (n = 4 mice per group; 10 measurements per image). **C**, **D** Representative z-stack immunofluorescence images showing ADAM17 expression in lung tissue under the indicated conditions (original magnification ×63). Data are presented as mean ± SD. Statistical analysis was performed using one-way ANOVA with appropriate post hoc testing, where appropriate, the Kruskal–Wallis test. *P < 0.05, **P < 0.01, ***P < 0.001, ****P < 0.0001

ADAM17 expression was quantified in murine lung tissue after LPS exposure. Under physiological conditions, vehicle-treated ADAM17^fl/fl^ Tie2-Cre^−^ and ADAM17^fl/fl^ Tie2-Cre^+^ knockout mice showed no differences in pulmonary ADAM17 mRNA expression (Fig. 1A). Three hours after LPS inhalation, ADAM17 mRNA levels in the lungs of ADAM17^fl/fl^ Tie2-Cre^+^ knockout mice were reduced by 77.5% compared to controls, reflecting effective knockout efficiency. At the protein level, statistical analysis of ADAM17 expression in lung tissue (Fig. 1B), along with representative z-stack immunofluorescence images (Fig. 1C–D), confirmed a significant reduction in ADAM17 protein in knockout mice 24 h after LPS inhalation.

These results highlight the pivotal contribution of endothelial-derived ADAM17 to total pulmonary expression. The endothelial-specific deletion was validated by flow cytometry (Supplementary Fig. 2). ADAM17 surface expression on pulmonary endothelial cells was quantified by mean fluorescence intensity (MFI) (Supplementary Fig. 2A). In ADAM17^fl/fl^ Tie2-Cre^+^ mice, endothelial ADAM17 surface levels were significantly reduced compared to ADAM17^fl/fl^ Tie2-Cre^−^ controls, confirming efficient targeting of endothelial cells. In contrast, ADAM17 surface expression on neutrophils and platelets was not reduced across blood, lung, and BAL fluid (Supplementary Fig. 2B, C). Importantly, ADAM17 expression on endothelial cells was selectively reduced, confirming the endothelial specificity of the genetic deletion.

### Endothelial ADAM17 promotes vascular barrier disruption during lung injury

**Fig. 2:**
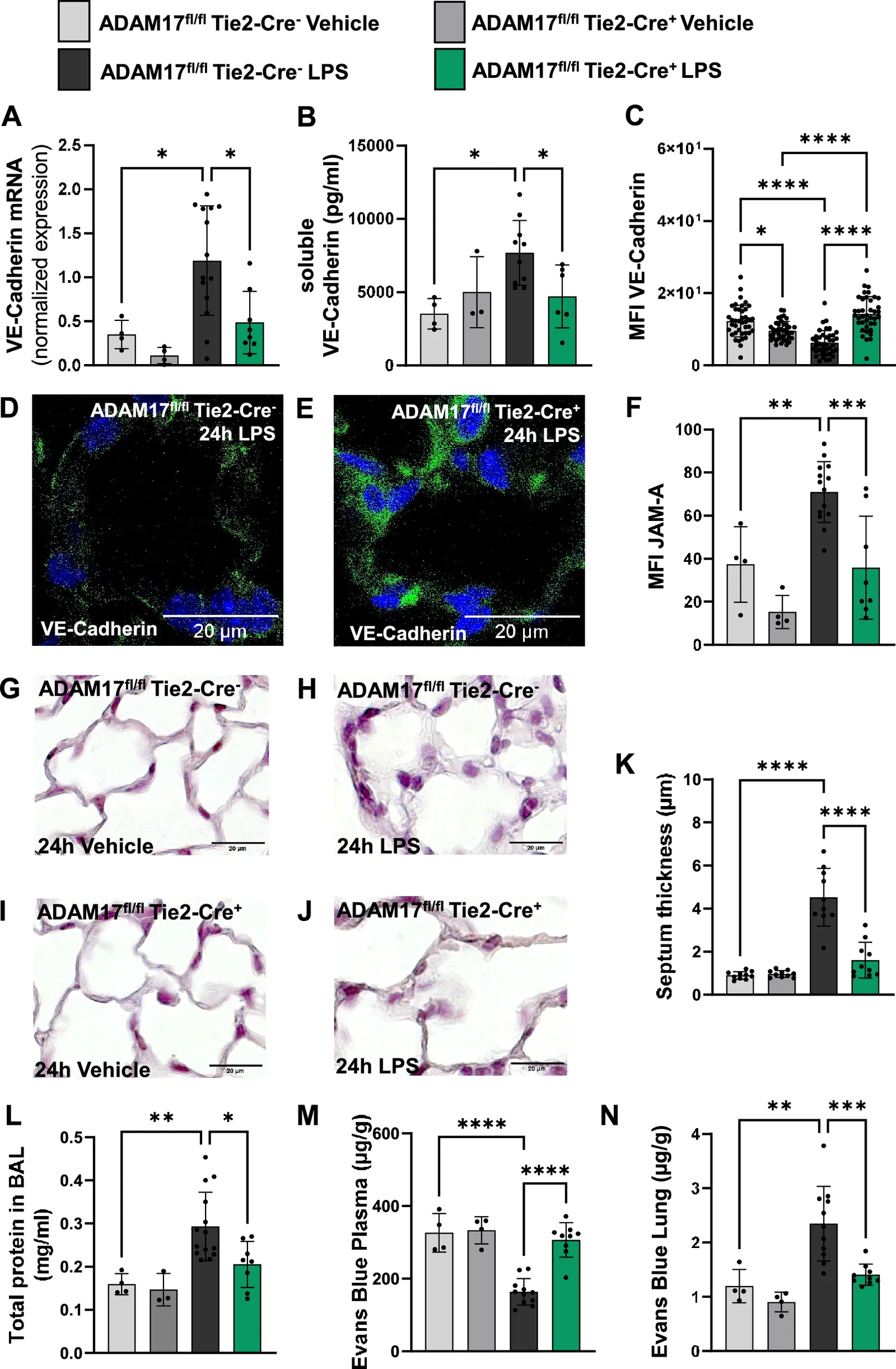
Endothelial ADAM17 promotes endothelial barrier disruption during lung injury. **A** Gene expression of vascular endothelial (VE)-cadherin in lung tissue three hours after LPS inhalation, assessed by RT-qPCR (n = 4/4/14/8 mice per group). **B** Quantification of soluble VE-cadherin in bronchoalveolar lavage (BAL) fluid three hours after LPS inhalation by ELISA (n = 4/3/10/6). **C** Quantification of VE-cadherin MFI in lung tissue using ImageJ (n = 4 mice per group; 10 measurements per image). **D**, **E** Representative immunofluorescence images of VE-cadherin expression (green) in murine lung tissue acquired using the LasX (n=4 mice per group). The representative VE-cadherin images shown in these panels depict the same magnified alveolar regions as those presented in Supplementary Fig. 3 and are reproduced here to facilitate comparison with the corresponding quantitative analysis. **F** Flow cytometric quantification of junctional adhesion molecule-A (JAM-A) MFI on pulmonary endothelial cells 24 h after LPS inhalation (n = 4/4/14/8). **G**–**J** Representative histological images illustrating alveolar septal thickness under the indicated conditions (original magnification ×20). **K** Quantification of alveolar septal thickness using ImageJ (n = 4 mice per group; 10 measurements per group). **L** Total protein concentration in BAL fluid 24 h after LPS inhalation (n = 4/3/14/8). **M**, **N** Photometric quantification of Evans blue in plasma (**M**) and lung tissue (**N**) six hours after LPS inhalation (n = 4/4/11/9). Data are presented as mean ± SD. Statistical analysis was performed using one-way ANOVA with appropriate post hoc testing, where appropriate, the Kruskal–Wallis test. *P < 0.05, **P < 0.01, ***P < 0.001, ****P < 0.0001

To investigate the role of endothelial ADAM17 in vascular permeability, we assessed endothelial junction integrity and lung barrier function following LPS exposure using multiple complementary assays (Fig. 2). These included analysis of VE-cadherin mRNA and protein (Fig. 2A–E), JAM-A surface expression (Fig. 2F), histological assessment of septal thickness (Fig. 2G–K), total protein levels in BAL fluid (Fig. 2L), and Evans blue extravasation (Fig. 2M-N).

Three hours after LPS inhalation, ADAM17^fl/fl^ Tie2-Cre^−^ mice showed increased VE-cadherin mRNA expression (Fig. 2A) and elevated soluble VE-cadherin (sVE-cadherin) in BAL fluid (Fig. 2B). In contrast, ADAM17^fl/fl^ Tie2-Cre^+^ knockout mice exhibited reduced VE-cadherin mRNA expression, diminished sVE-cadherin release, preserved endothelial VE-cadherin surface expression (Fig. 2C), and fewer intercellular gaps (Fig. 2D-E and Supplementary Fig. 3A–D), consistent with improved preservation of endothelial junctional integrity.

JAM-A surface expression on pulmonary endothelial cells was quantified by flow cytometry 24 h after LPS exposure. LPS significantly increased JAM-A mean fluorescence intensity (MFI) in control mice, whereas endothelial ADAM17 deficiency markedly reduced JAM-A surface expression (Fig. 2F). To further assess endothelial junctional integrity, we analyzed the junctional proteins ZO-1, occludin, and claudin-5 by immunofluorescence. 24 h after LPS inhalation, ADAM17fl/fl Tie2-Cre− mice exhibited significantly reduced surface expression of ZO-1 and occludin (Supplementary Fig. 4) as well as claudin-5 (Supplementary Fig. 5) compared with vehicle-treated controls. Pharmacological inhibition of ADAM17 using TAPI-1 or GW280264X, as well as endothelial-specific ADAM17 deficiency, significantly preserved the surface expression of all three junctional proteins following LPS exposure, indicating improved endothelial junctional integrity. Consistent with these in vivo findings, immunofluorescence analyses of HMEC-1 endothelial cells (Supplementary Fig. 6) and H441 epithelial cells (Supplementary Fig. 7) demonstrated that LPS stimulation for 3 h markedly reduced the surface expression of ZO-1, occludin, and claudin-5, whereas treatment with TAPI-1 or GW280264X restored the expression of all three proteins.

Lung histology demonstrated substantial architectural preservation in knockout mice. Mayer’s hematoxylin staining revealed significantly reduced septal thickness compared to controls (Fig. 2K), supported by representative images (Fig. 2G–J). Total protein concentration in BAL fluid, measured 24 h after LPS inhalation, was significantly lower in knockout mice compared to controls, indicating reduced alveolar-capillary leakage (Fig. 2L).

Finally, we quantified microvascular leakage using the Evans blue extravasation assay six hours after LPS exposure, corresponding to the peak leakage time in murine acute lung injury [2]. We observed reduced plasma Evans blue concentrations in control but not in knockout mice (Fig. 2M), while knockout lungs showed significantly less Evans blue accumulation (Fig. 2N), indicating preserved endothelial barrier function.

### Endothelial ADAM17 enhances neutrophil migration into the inflamed lung

**Fig. 3:**
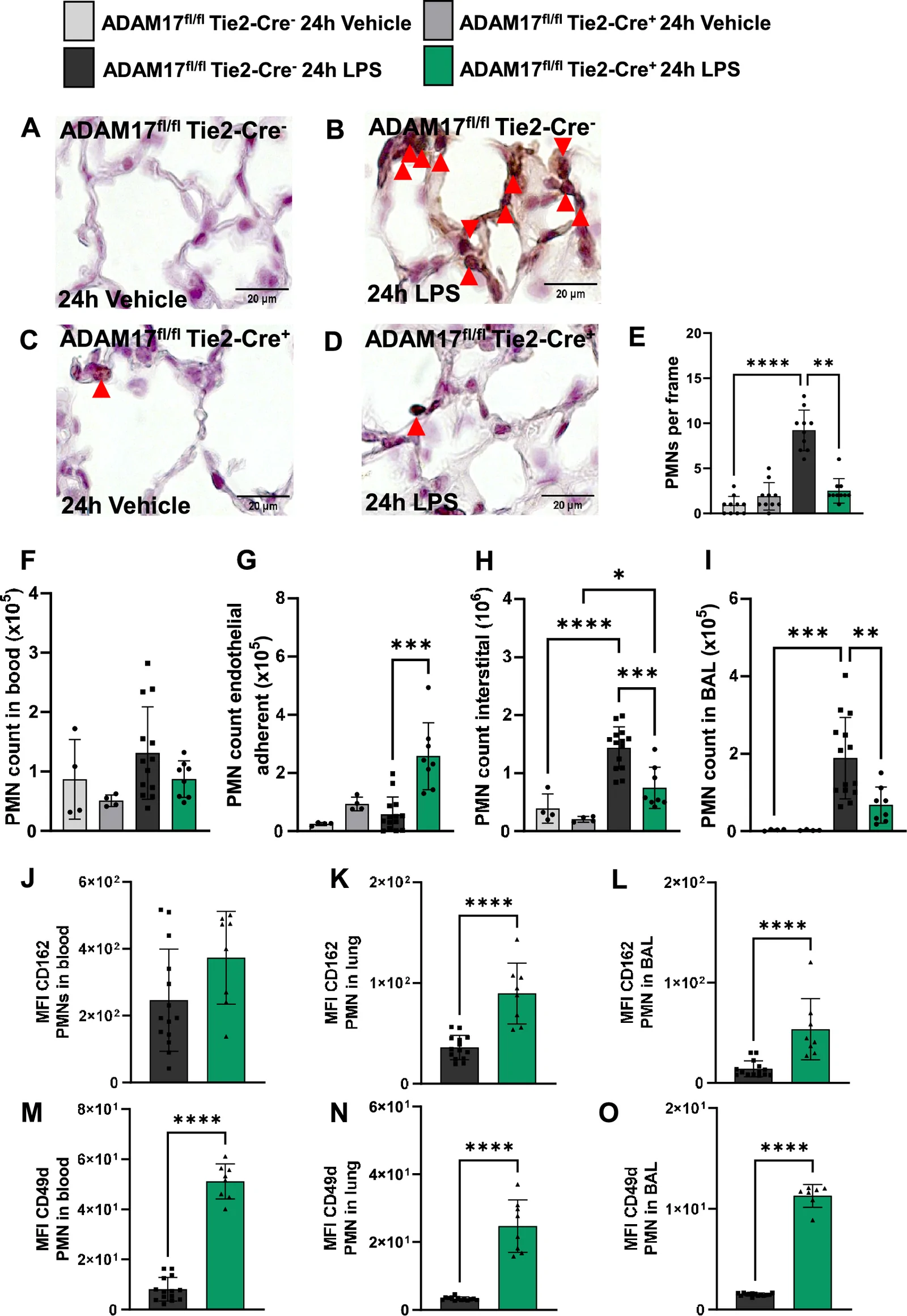
Endothelial ADAM17 enhances neutrophil migration into the inflamed lung. **A**–**D** Representative immunohistochemical images showing polymorphonuclear neutrophil (PMN) distribution in lung tissue under the indicated conditions. PMNs were stained using an anti-Ly6G/C antibody (brown) with hematoxylin counterstain. Red arrows indicate PMNs (n = 4 mice per group; original magnification ×20). **E** Quantification of PMNs per high-power field (HPF; n = 4 mice per group, 10 images per group). **F**–**I** Flow cytometric quantification of PMNs in blood (**F**), adherent to the pulmonary endothelium (**G**), within the interstitium (**H**), and in BAL fluid (**I**) 24 h after LPS inhalation (n = 4/4/12–14/8). **J**–**L** Quantification of CD162 (PSGL-1) MFI and (**M**–**O**) CD49d MFI on PMNs isolated from blood, lung, and BAL. Data are presented as mean ± SD. Statistical analysis was performed using one-way ANOVA with appropriate post hoc testing, where appropriate, the Kruskal–Wallis test. *P < 0.05, **P < 0.01, ***P < 0.001, ****P < 0.0001

Next, we investigated how endothelial ADAM17 affects neutrophil infiltration during LPS-induced lung inflammation. Representative immunohistochemical images (Fig. 3A–D) show neutrophils, identified as brown-stained cells (red arrows), migrating into lung tissue. Quantification revealed a significantly lower number of neutrophils in the lungs of ADAM17^fl/fl^ Tie2-Cre^+^ knockout mice compared to ADAM17^fl/fl^ Tie2-Cre^−^ controls (Fig. 3E).

To further dissect neutrophil migration across pulmonary compartments, we performed compartment-specific flow cytometry analysis 24 h after LPS exposure, corresponding to peak transendothelial and transepithelial migration [2]. We quantified neutrophils in blood (Fig. 3F), adhered to the pulmonary endothelium (Fig. 3G), within the interstitium (Fig. 3H), and in the alveolar space (Fig. 3I). Circulating neutrophil counts were similar between knockout and control mice (Fig. 3F). However, knockout mice showed significantly more neutrophils retained at the vascular endothelium (Fig. 3G) and significantly fewer neutrophils in the interstitium (Fig. 3H) and alveolar space (Fig. 3I), consistent with altered neutrophil passage across the endothelial barrier.

To further investigate whether ADAM17 directly contributes to neutrophil transmigration, we performed an in vitro transmigration assay using primary human neutrophils migrating across an endothelial–epithelial Transwell barrier (Supplementary Fig. 8). Stimulation with fMLP markedly increased neutrophil transmigration compared with unstimulated controls. Pharmacological inhibition of ADAM17 with TAPI-1 or combined ADAM10/17 inhibition using GW280264X significantly reduced neutrophil transmigration. In parallel, ADAM17 inhibition attenuated fMLP-induced MPO release and reduced the expression of the transmigration-associated adhesion molecules CD11b and JAM-A on transmigrated neutrophils. These findings provide complementary mechanistic evidence supporting a role for ADAM17 in neutrophil transmigration.

We next assessed whether endothelial ADAM17 affects neutrophil adhesion molecule expression. Flow cytometry analysis of PMNs in blood, lung, and BAL fluid revealed significantly elevated surface expression of CD162 (Figs. 3J–L) and CD49d (Figs. 3M–O) in knockout mice. CD162 was increased in lung and BAL PMNs, while CD49d expression was significantly upregulated in all compartments compared to controls. This upregulation of CD162 and CD49d correlates with the enhanced retention of PMNs at the endothelium and reduced tissue infiltration.

Together, these findings support a role for endothelial ADAM17 in regulating neutrophil trafficking into inflamed lung tissue through effects on endothelial barrier function and neutrophil transmigration.

### Endothelial ADAM17 selectively amplifies cytokine receptor signaling

**Fig. 4:**
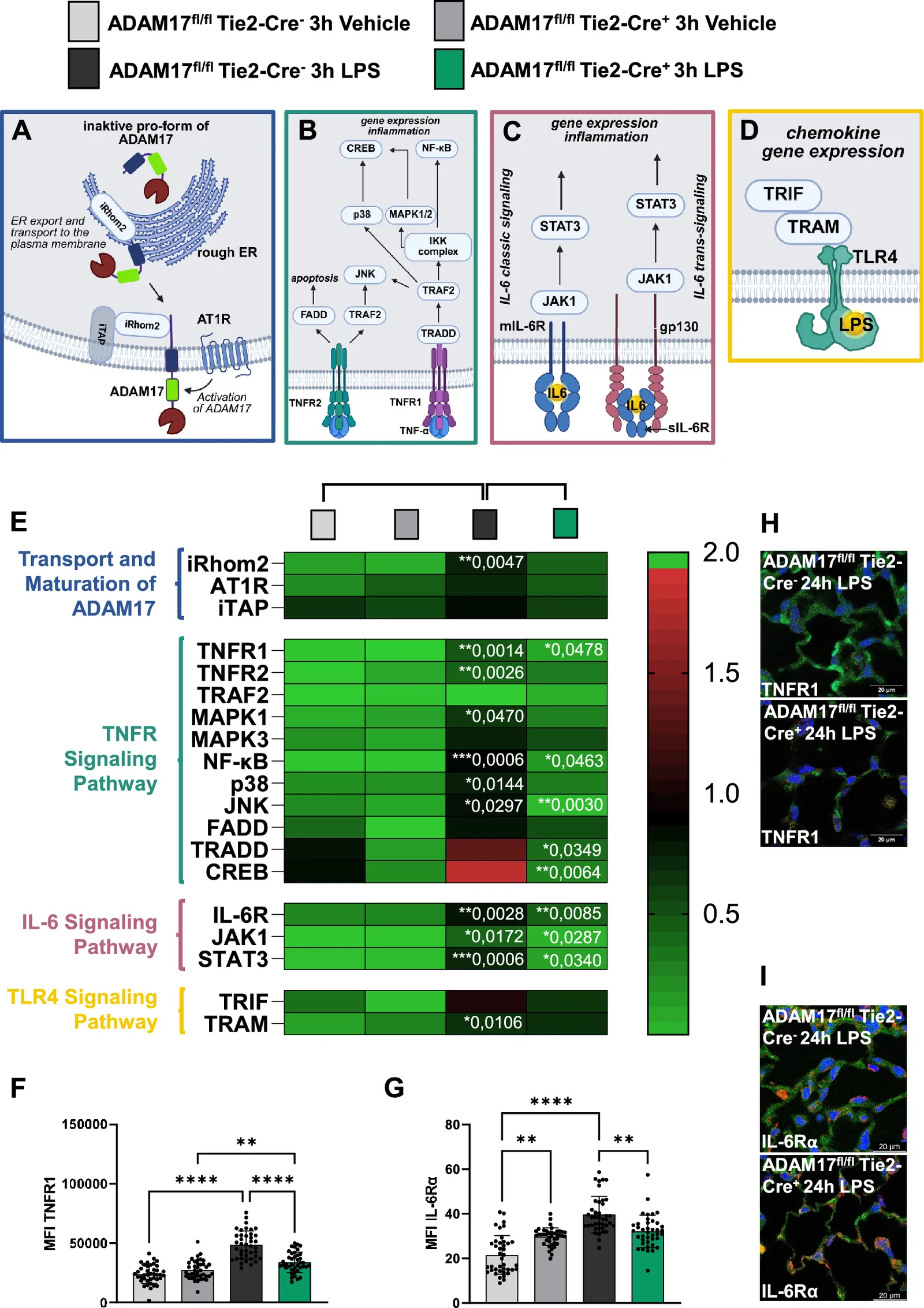
Endothelial ADAM17 amplifies inflammatory TNFR1 and IL-6R signaling pathways in acute lung injury. **A** Schematic overview of ADAM17 maturation and intracellular transport. **B**–**D** Schematic representations of TNFR1/2 (**B**), IL-6R (**C**), and TLR4 (**D**) signaling pathways. **E** RT-qPCR-based gene expression analysis of inflammatory signaling components in murine lung tissue three hours after LPS inhalation (n = 4/4/10–14/6–8). **F**–**G** Quantification of TNFR1 (**F**) and IL-6Rα (**G**) MFI in lung tissue using LasX (n = 4 mice per group; 10 measurements per image). **H**–**I** Representative immunofluorescence images of TNFR1 (**H**) and IL-6Rα (**I**) expression (green) in murine lung tissue acquired using the LasX (n=4 mice per group). The representative images shown in these panels depict the same magnified regions as those presented in Supplementary Fig. 10 and are reproduced here to facilitate comparison with the corresponding quantitative analysis. Data are presented as mean ± SD; Statistical analysis was performed using one-way ANOVA with appropriate post hoc testing, where appropriate, the Kruskal–Wallis test. *P < 0.05, **P < 0.01, ***P < 0.001, ****P < 0.0001

We next examined whether endothelial ADAM17 regulates inflammatory signaling pathways during LPS-induced lung inflammation. Using gene expression analysis, we assessed ADAM17 maturation and transport factors, TNFR and IL-6R signaling cascades, and TLR4-dependent pathways (Fig. 4).

Three hours after LPS inhalation, expression of the ADAM17 regulatory proteins iRhom2, AT1R, and iTAP increased similarly in control and knockout mice, indicating that endothelial ADAM17 does not influence ADAM17 maturation or trafficking (Fig. 4A).

In contrast, endothelial ADAM17 deletion selectively attenuated proinflammatory receptor signaling. Knockout mice showed a significant reduction in TNFR1 mRNA expression compared to controls, while TNFR2 expression remained unchanged (Fig. 4B). Consistent with impaired TNFR1 signaling, transcript levels of downstream mediators Nf-κB, JNK, TRADD, and CREB were significantly reduced. Endothelial ADAM17 deletion also significantly decreased IL-6R expression, accompanied by reduced induction of the downstream signaling molecules JAK1 and STAT3 (Fig. 4C).

By contrast, LPS activated the TLR4 pathway independently of endothelial ADAM17. Expression of the adaptor molecules TRIF and TRAM increased after LPS exposure to a similar extent in knockout and control mice, indicating preserved TLR4 signaling (Fig. 4D). To determine the cellular localization of TLR4, we performed co-immunofluorescence staining of TLR4 with the endothelial marker vWF, the epithelial marker F-actin, and the neutrophil marker Ly6G/C (Supplementary Fig. 9). 24 h after LPS inhalation, TLR4 expression was markedly increased in both ADAM17^fl/fl^ Tie2-Cre^−^ and ADAM17^fl/fl^ Tie2-Cre^+^ mice compared with vehicle-treated controls. TLR4 immunoreactivity was detected in pulmonary endothelial cells, alveolar epithelial cells, and infiltrating Ly6G/C-positive neutrophils within the alveolar space, with no apparent qualitative differences between genotypes, confirming that TLR4 activation occurs independently of endothelial ADAM17.

We confirmed the transcriptional findings of Fig. 4E at the protein level. Immunofluorescence analysis 24 h after LPS inhalation revealed significantly reduced expression of TNFR1 and IL-6Rα in lung tissue of knockout mice compared to controls (Supplementary Fig. 10; Fig. 4F–I). Notably, under basal conditions, knockout mice exhibited higher IL-6Rα expression than controls, suggesting altered receptor homeostasis in the absence of endothelial ADAM17.

Together, these data demonstrate that endothelial ADAM17 selectively amplifies TNFR1- and IL-6R-dependent inflammatory signaling while leaving ADAM17 maturation and TLR4 activation intact, positioning endothelial ADAM17 as a key modulator of cytokine-driven amplification loops in acute pulmonary inflammation.

### Endothelial ADAM17 amplifies alveolar inflammatory mediator release

**Fig. 5:**
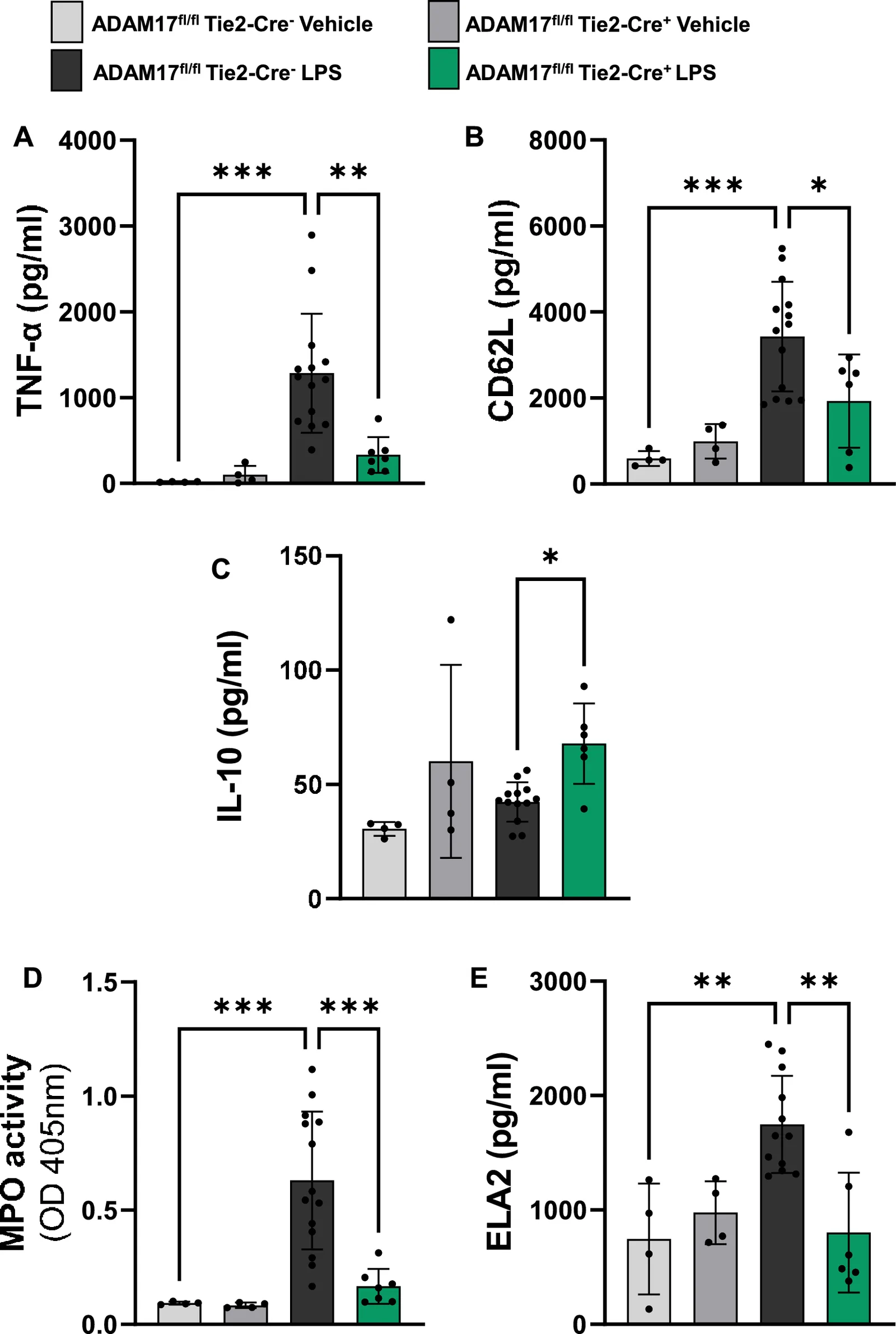
Endothelial ADAM17 increases alveolar inflammation during acute lung injury. Quantification of (**A**) TNF-α, (**B**) soluble L-selectin (CD62L), (**C**), Interleukin-10 (IL-10) three hours after LPS inhalation in bronchoalveolar lavage (BAL) by ELISA. Evaluation of neutrophil activity by (**D**) semiquantitative analysis of myeloperoxidase (MPO) and (**E**) quantification of neutrophil elastase (ELA2) by ELISA 24 h after LPS exposure in BAL fluid. Data are presented as mean ± SD (n=4/4/12–14/6–7); Individual analytes were analyzed separately using one-way ANOVA with appropriate post hoc testing, where appropriate, the Kruskal–Wallis test. *P < 0.05, **P < 0.01, ***P < 0.001, ****P < 0.0001, using one-way ANOVA

We next examined how endothelial ADAM17 influences alveolar inflammation by quantifying cytokines and neutrophil-derived mediators in bronchoalveolar lavage (BAL) fluid following LPS exposure (Fig. 5).

Three hours after LPS inhalation, control mice exhibited a pronounced increase in TNF-α levels in BAL fluid, whereas endothelial ADAM17 deletion significantly reduced TNF-α release (Fig. 5A). This reduction indicates impaired shedding of membrane-bound TNF-α and attenuated generation of soluble TNF-α in the absence of endothelial ADAM17.

LPS stimulation also markedly increased the release of soluble CD62L in control mice, while knockout mice showed significantly lower sCD62L levels in BAL fluid (Fig. 5B). Reduced CD62L shedding coincided with enhanced retention of neutrophils at the vascular endothelium observed in vivo, consistent with impaired transmigration.

In contrast, knockout mice displayed significantly higher levels of the anti-inflammatory cytokine IL-10 following LPS exposure compared to controls, indicating a shift toward an anti-inflammatory alveolar milieu (Fig. 5C).

Markers of neutrophil activation further supported this protective phenotype. Control mice showed robust increases in myeloperoxidase (MPO) and neutrophil elastase (ELA2) in BAL fluid 24 h after LPS inhalation, whereas endothelial ADAM17 deletion markedly attenuated the release of both enzymes (Fig. 5D–E).

Together, these findings demonstrate that endothelial ADAM17 promotes alveolar inflammation by enhancing the release of proinflammatory cytokines, facilitating CD62L shedding, and driving neutrophil activation, while its deletion limits alveolar tissue injury and favors an anti-inflammatory environment.

### Systemic ADAM17 inhibition attenuates LPS-induced lung inflammation

**Fig. 6:**
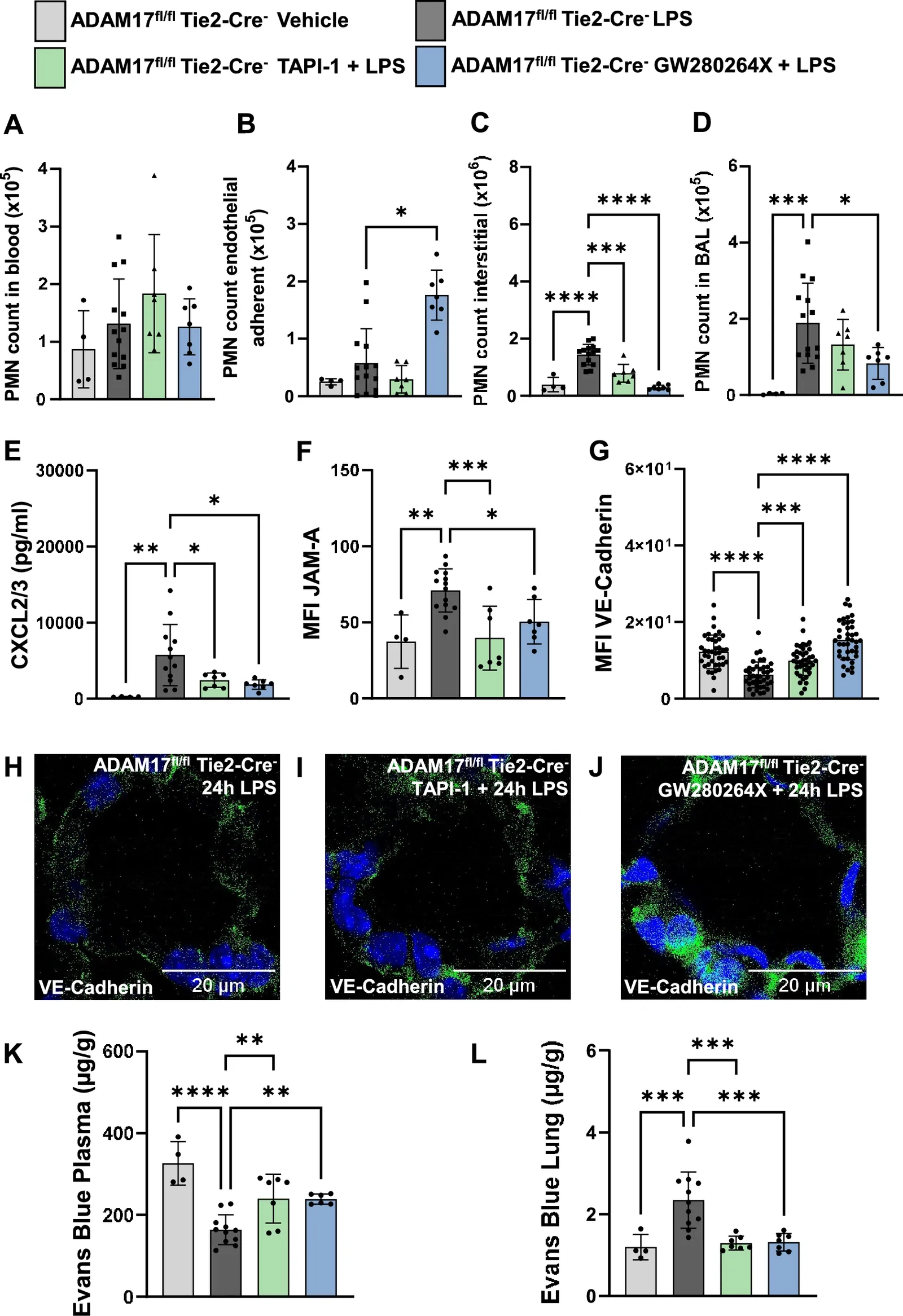
Systemic ADAM17 inhibition reduces LPS-induced lung inflammation. **A**–**D** Flow cytometric analysis of PMN distribution across lung compartments: blood (**A**), endothelial-adherent (**B**), interstitial (**C**), and BAL (**D**) 24 h after LPS inhalation following pharmacological ADAM17 inhibition administered 1 h before LPS exposure (n = 4/13–14/7/7). **E** Quantification of CXCL2/3 in BAL fluid three hours after LPS inhalation by ELISA (n = 4/12/7/7). **F** Flow cytometric quantification of JAM-A MFI on pulmonary endothelial cells 24 h after LPS inhalation (n = 4/14/7/7). **G** Quantification of VE-cadherin MFI in lung tissue using LasX (n = 4 mice per group; 10 measurements per image). **(H-J)** Representative immunofluorescence images of VE-cadherin expression (green) in murine lung tissue acquired using the LasX (n=4 mice per group). The representative VE-cadherin images shown in these panels depict the same magnified alveolar regions as those presented in Supplementary Fig. 3 and are reproduced here to facilitate comparison with the corresponding quantitative analysis. **K**, **L** Photometric quantification of Evans blue in plasma (K) and lung tissue (L) six hours after LPS inhalation (n = 4/11/7/6–7). The data shown for ADAM17^fl/fl^ Tie2-Cre^−^ controls were previously presented in the following panels: Fig. 6A–D in Fig. 3F–I, Fig. 6F in Fig. 2F, Fig. 6G in Fig. 2C, and Fig. 6K–L in Fig. 2M–N (to minimize animal numbers in accordance with the 3R principle). Data are presented as mean ± SD; Statistical analysis was performed using one-way ANOVA with appropriate post hoc testing, where appropriate, the Kruskal–Wallis test. *P < 0.05, **P < 0.01, ***P < 0.001, ****P < 0.0001

We next investigated whether pharmacological inhibition of ADAM17 recapitulates the protective phenotype observed in endothelial-specific knockout mice. We analyzed neutrophil migration across pulmonary compartments 24 h after LPS inhalation following systemic inhibition with TAPI-1 or GW280264X (Fig. 6).

LPS exposure markedly increased neutrophil migration from the circulation into the lung interstitium and alveolar space (Fig. 6A–D). TAPI-1 treatment significantly reduced neutrophil accumulation in the interstitium, while GW280264X treatment decreased neutrophil numbers in both the interstitium and alveoli. Combined inhibition of ADAM17 and ADAM10 further potentiated this effect, suggesting additive or synergistic roles of these proteases. Notably, dual inhibition led to a significant accumulation of neutrophils at the pulmonary endothelium, indicating impaired transendothelial migration and retention of immune cells at the vascular wall.

Systemic ADAM17 inhibition also attenuated inflammatory signaling and endothelial junction remodeling. To determine whether systemic ADAM17 inhibition additionally modulates neutrophil-attracting chemokine signals within the alveolar compartment, we quantified CXCL2/3 in BAL fluid. Pre-treatment with TAPI-1 or GW280264X significantly reduced LPS-induced release of the neutrophil-attracting chemokines CXCL2/3 (Fig. 6E). LPS increased JAM-A surface expression on pulmonary endothelial cells, whereas inhibition of ADAM17 alone or in combination with ADAM10 significantly reduced JAM-A mean fluorescence intensity (Fig. 6F). In parallel, LPS reduced VE-cadherin expression and increased intercellular gap formation, whereas systemic inhibition of ADAM17 was associated with preserved VE-cadherin expression, reduced gap formation, and improved endothelial junction integrity (Fig. 6G-J; Supplementary Fig. 3).

Finally, we assessed microvascular permeability using the Evans blue extravasation assay six hours after LPS exposure. LPS significantly increased Evans blue accumulation in lung tissue, whereas systemic ADAM17 inhibition markedly reduced dye extravasation (Fig. 6L). Consistent with reduced vascular leakage, plasma Evans blue concentrations increased following ADAM17 inhibition compared to LPS-treated controls (Fig. 6K).

Together, these data demonstrate that systemic ADAM17 inhibition attenuates key pathophysiological hallmarks of ARDS, including excessive neutrophil recruitment, endothelial barrier disruption, and vascular leakage, thereby supporting ADAM17 as a rational therapeutic target in acute lung injury.

## Discussion

Building on and substantially extending previous work by Dreymueller et al., our findings support a central role for endothelial ADAM17 in regulating neutrophil recruitment, endothelial barrier integrity, and inflammatory signal amplification during LPS-induced acute pulmonary inflammation. By combining endothelial-specific genetic deletion with systemic pharmacological inhibition, we provide mechanistic evidence that ADAM17 expressed by the pulmonary endothelium drives key pathophysiological hallmarks of ARDS, including excessive neutrophil transmigration, junctional destabilization, vascular leakage, and alveolar inflammation. Together, our findings highlight endothelial ADAM17 as a critical inflammatory amplifier and support its therapeutic targeting to preserve vascular barrier integrity and limit acute lung injury.

LPS exposure induced a pronounced upregulation of ADAM17 mRNA and protein expression in lung tissue. Although the transcriptional regulation of ADAM17 during acute pulmonary inflammation remains incompletely understood, increased expression likely represents an additional layer of inflammatory control beyond post-translational activation. Elevated ADAM17 levels can amplify inflammation by promoting the release of cytokines, cytokine receptors, and adhesion molecules. Consistent with our findings, ADAM17 expression is increased in chronic inflammatory lung diseases such as asthma and COPD [11], and LPS augments both ADAM17 transcription and shedding activity in endothelial cells [12]. Notably, LPS selectively increased ADAM17 but not ADAM10 transcription, reinforcing the dominant role of ADAM17 in endotoxin-driven inflammation [12].

To specifically dissect the endothelial contribution, we employed the conditional ADAM17^fl/fl^ Tie2-Cre^+^ mouse model. Endothelial ADAM17 deletion reduced pulmonary ADAM17 mRNA expression by 77.5%, confirming that endothelial cells constitute a major source of ADAM17 in inflamed lung tissue. These findings are consistent with previous reports in Tie2-adam17^−/−^ mice [12]. Although the Tie2 promoter has been debated for activity in hematopoietic cells [13], extensive characterization has demonstrated endothelial-restricted deletion of ADAM17 with preserved expression in peripheral blood cells and alveolar macrophages [12, 14]. Our flow cytometric validation further confirmed intact ADAM17 expression in PMNs and platelets, supporting endothelial specificity.

Endothelial ADAM17 exerted profound effects on endothelial barrier regulation and microvascular permeability. In control mice, LPS caused severe architectural lung damage, septal thickening, and protein-rich pulmonary oedema, whereas endothelial ADAM17 deficiency preserved lung structure and barrier integrity. These findings corroborate and substantially extent earlier observations by Dreymueller et al., who demonstrated protection from endotoxin-induced lung injury in Tie2-adam17^−/−^ mice, including reduced alveolar protein accumulation, preservation of lung architecture, and diminished interalveolar septal thickening following LPS challenge [12]. Whereas their study primarily established a protective role of endothelial ADAM17 deficiency at the histological and physiological level, our findings provide additional mechanistic insight into how endothelial ADAM17 contributes to vascular barrier failure during acute lung inflammation.

At the molecular level, endothelial ADAM17 regulated key junctional proteins. JAM-A, a critical tight-junction component under physiological conditions [15], redistributes to the apical endothelial surface during inflammation to facilitate leukocyte transmigration [16, 17]. This redistribution and increased JAM-A surface expression were absent in endothelial ADAM17-deficient mice. Previous studies demonstrated that LPS induces junctional disruption through shedding of JAM-A and cadherins [18], a process attenuated by ADAM17 inhibition [12, 19]. Notably, Dreymueller et al. reported reduced release of soluble JAM-A into BAL fluid following endothelial ADAM17 deletion, suggesting impaired junctional remodeling in the absence of ADAM17 [12]. Our findings build directly upon these observations by demonstrating altered endothelial JAM-A surface expression in vivo, thereby linking endothelial ADAM17 to the regulation of junctional organization and leukocyte-permissive endothelial phenotypes during acute pulmonary inflammation. Beyond JAM-A, our additional analyses demonstrate that endothelial ADAM17 activity is associated with a broader disruption of endothelial junctional architecture. In vivo, endothelial ADAM17 deficiency as well as pharmacological ADAM17 inhibition preserved the surface expression of the tight junction proteins ZO-1, occludin, and claudin-5 following LPS challenge, suggesting improved maintenance of endothelial barrier integrity. These findings were corroborated in HMEC-1 endothelial and H441 epithelial cells, where ADAM17 inhibition similarly restored junctional protein expression after LPS stimulation. While, in contrast to JAM-A, ZO-1, occludin, and claudin-5 are not established direct substrates of ADAM17, their preservation likely reflects reduced inflammatory junctional remodeling secondary to ADAM17 inhibition. Together, these findings indicate that ADAM17 contributes not only to JAM-A regulation but also to a broader impairment of junctional organization during acute lung injury.

VE-Cadherin represents another important component of the endothelial adherens junction that was preserved in endothelial deficient mice. Although ADAM10 is widely recognized as the principal VE-Cadherin sheddase [19, 20], endothelial ADAM17 deficiency in our model was associated with reduced soluble VE-cadherin levels in BAL fluid and preserved endothelial VE-cadherin expression. Together with the concomitant preservation of the tight junction proteins ZO-1, occludin and claudin-5, these findings are consistent with an overall preservation of endothelial junctional integrity during acute pulmonary inflammation. However, the present data do not distinguish between direct regulation of VE-cadherin and indirect preservation secondary to the reduced inflammatory response observed in endothelial ADAM17-deficient mice.

Functional permeability assays supported these structural findings. LPS-induced increases in BAL protein concentration and Evans blue extravasation were abolished in endothelial ADAM17-deficient mice. Consistent with our observations, Dreymueller et al. reported reduced BAL protein levels and improved lung histology in Tie2-adam17−/− mice following endotoxin exposure [12]. However, their study primarily assessed barrier dysfunction using BAL protein measurements and wet-to-dry lung weight ratio, which provide indirect measures of alveolar-capillary permeability. While BAL protein content alone incompletely reflects barrier function [21], direct assessment of vascular permeability by Evans blue extravasation provides compelling evidence that endothelial ADAM17 critically governs microvascular leakage. To our knowledge, this represents the first direct demonstration of reduced Evans blue extravasation in ADAM17^fl/fl^ Tie2-Cre^+^ mice and thereby provides direct functional confirmation of the barrier-protective phenotype previously inferred from histological and BAL-based analyses.

Endothelial ADAM17 also emerged as a key regulator of neutrophil trafficking. Endothelial ADAM17 deficiency caused pronounced retention of PMNs at the vascular endothelium, accompanied by reduced accumulation within the interstitial and alveolar compartments and increased expression of CD49d and PSGL-1. These findings corroborate and substantially extend earlier observations by Dreymueller et al., who reported significantly reduced leukocyte and neutrophil recruitment into the bronchoalveolar compartment of Tie2-adam17−/− mice following LPS challenge [12]. Pharmacological inhibition of ADAM17 similarly attenuated pulmonary neutrophil accumulation, further supporting a role for ADAM17-dependent shedding processes in regulating inflammatory cell recruitment. However, because previous studies relied primarily on total lung and BAL cell counts, the precise step within the leukocyte recruitment cascade remained unresolved. Our compartment-specific analysis extends these observations by demonstrating that endothelial ADAM17 deficiency does not simply reduce overall neutrophil recruitment but is associated with increased neutrophil retention at the endothelial surface and reduced accumulation within the interstitial and alveolar compartments. Importantly, complementary in vitro transmigration experiments demonstrated that pharmacological ADAM17 inhibition significantly reduced neutrophil transmigration across an endothelial-epithelial barrier, supporting a role for ADAM17 in neutrophil transmigration.

Mechanistically, Dreymueller et al. demonstrated reduced shedding of endothelial adhesion and signaling molecules, including JAM-A and CX3CL1, in Tie2-adam17-/- mice [12]. Our findings are consistent with these observations and further suggest that endothelial ADAM17 regulates the adhesive state of transmigrating neutrophils. The increased surface expression of neutrophil CD49d and PSGL-1 observed in endothelial ADAM17-deficient mice is consistent with the enhanced endothelial retention of neutrophils. In line with these observations, pharmacological ADAM17 inhibition reduced neutrophil transmigration in vitro and was accompanied by reduced fMLP-induced MPO release as well as decreased expression of the transmigration-associated molecules CD11b and JAM-A. Together, these findings support a role for ADAM17 in regulating neutrophil trafficking during acute pulmonary inflammation. Our observations are in line with previous reports demonstrating that ADAM family proteases regulate the surface availability of key leukocyte adhesion molecules. For example, ADAM17 inhibition preserved PSGL-1 surface expression in apoptotic CEM cells, while ADAM10 deficiency prevented cytokine-induced loss of CD162 in neutrophil-like HL-60 cells [22, 23]. ADAM17 cleaves multiple endothelial adhesion molecules, including ICAM-1, VCAM-1, JAM-A, and PECAM-1, thereby regulating adhesion–deadhesion dynamics [24]. In contrast to endothelial deletion models, most studies investigating ADAM17-dependent leukocyte trafficking have focused on hematopoietic ADAM17 and neutrophil-expressed substrates such as L-selectin [10, 25–27]. Indeed, leukocyte-specific ADAM17 deficiency has been shown to alter neutrophil recruitment kinetics through impaired L-selectin shedding rather than endothelial barrier regulation [25]. Importantly, our previous in vitro adhesion studies consistently demonstrated increased rather than decreased neutrophil adhesion following dual ADAM17/10 inhibition with GW280264X [10], arguing against impaired initial adhesion as the primary explanation for the reduced extravascular neutrophil accumulation observed in endothelial ADAM17-deficient mice. Collectively, our findings support a distinct role of endothelial ADAM17 in regulating neutrophil compartmental trafficking through coordinated regulation of inflammatory signaling, endothelial barrier function, neutrophil adhesion dynamics, and transmigration.

At the signaling level, endothelial ADAM17 selectively amplified TNFR1 and IL-6R pathways. Endothelial ADAM17 deletion reduced TNFR1 gene and protein expression and attenuated downstream signaling via Nf-κB, JNK, TRADD, and CREB. ADAM17 is well established as the principal sheddase for TNF-α and TNFR1 [28, 29], and recent work implicates ADAM17-dependent TNFR1 shedding in endothelial necroptosis and transendothelial migration [30]. The reduced TNFR1 expression observed here likely reflects feedback inhibition secondary to diminished inflammatory signaling rather than direct transcriptional control. Importantly, while previous studies established a protective role of endothelial ADAM17 deficiency in acute lung injury, the impact of endothelial ADAM17 on downstream inflammatory receptor signaling remained largely unexplored [12]. Our findings therefore provide mechanistic evidence linking endothelial ADAM17 activity to TNFR1-dependent signal amplification during pulmonary inflammation.

Similarly, endothelial ADAM17 deletion attenuated IL-6R signaling, reducing IL-6R, JAK1, and STAT3 expression. ADAM17-mediated IL-6R shedding enables IL-6 trans-signaling [31–34], and diminished STAT3 activation in ADAM17-deficient settings has been reported previously [35]. Notably, Dreymueller et al. observed reduced inflammatory cytokine release in Tie2-adam17−/− mice but did not investigate the underlying receptor-mediated signaling pathways [12]. Our data extend these observations by identifying endothelial ADAM17 as a selective regulator of IL-6R/JAK1/STAT3 signaling and suggest that modulation of cytokine receptor availability represents an important mechanism through which endothelial ADAM17 amplifies pulmonary inflammation. Compared with leukocyte-specific ADAM17 deletion, which only modestly reduces alveolar IL-6R levels [25], our findings further underscore the dominant role of endothelial ADAM17 in pulmonary IL-6 signaling.

Endothelial ADAM17 further amplified alveolar inflammation by promoting release of TNF-α, soluble CD62L, MPO, and ELA2. These findings are consistent with previous observations by Dreymueller et al., who reported reduced alveolar levels of TNF-α, IL-6, and CX3CL1 in Tie2-adam17−/− mice and after pharmacological ADAM17 inhibition following LPS challenge [12]. Together, these studies support a central role for endothelial ADAM17 in shaping the inflammatory mediator milieu of the injured lung. Our findings substantially extend this concept by demonstrating that endothelial ADAM17 not only regulates cytokine release but also influences markers of neutrophil activation and turnover within the alveolar compartment.

Although TNF-α production was reduced, it was not abolished, consistent with contributions from epithelial cells and alveolar macrophages [36] and alternative proteases [37]. Notably, endothelial ADAM17 deletion reduced CD62L shedding, a process traditionally attributed to leukocyte-derived ADAM17 [38–40]. Our findings suggest that endothelial ADAM17 contributes to CD62L shedding during leukocyte–endothelial interactions, revealing a previously underappreciated regulatory axis.

Reduced MPO and ELA2 release further indicated attenuated neutrophil activation. Although these enzymes are released by neutrophils independently of endothelial ADAM17 activity, their reduction likely reflects diminished neutrophil recruitment and activation within the alveolar compartment. This interpretation is supported by the concomitant reduction in neutrophil transmigration observed in endothelial ADAM17-deficient mice. Consequently, endothelial ADAM17 may amplify pulmonary inflammation not only through cytokine receptor signaling and barrier disruption but also by promoting the accumulation and activation of effector neutrophils within the alveolar space. Such attenuation may limit excessive ROS production, NET formation, and tissue injury [41–43].

Importantly, systemic ADAM17 inhibition largely phenocopied the endothelial-specific knockout. Pharmacological inhibition reduced neutrophil recruitment, stabilized endothelial junctions, decreased chemokine release, and preserved vascular barrier integrity. Dual inhibition of ADAM17 and ADAM10 further enhanced these effects, suggesting cooperative protease activity. These findings are consistent with earlier observations by Dreymueller et al., who demonstrated protection from LPS-induced acute lung injury using endothelial ADAM17-deficient mice and the dual ADAM10/17 inhibitor GW280264X, resulting in reduced cytokine release, vascular permeability, oedema formation, and pulmonary leukocyte recruitment [12]. Importantly, because GW280264X inhibits both ADAM10 and ADAM17, the relative contribution of the individual proteases remained difficult to define. By additionally employing the ADAM17-selective inhibitor TAPI-1, our study provides further pharmacological evidence that many of the protective effects can be attributed specifically to ADAM17 inhibition, while the more pronounced responses observed with GW280264X suggest cooperative contributions of ADAM10-dependent shedding pathways. Despite these promising findings, several translational limitations should be considered. In the present study, pharmacological ADAM17 inhibition was administered prior to LPS challenge, as commonly performed in experimental target-validation studies to investigate the contribution to disease pathogenesis. Therefore, the therapeutic efficacy of ADAM17 inhibition after disease onset remains to be established. Moreover, given the broad substrate repertoire of ADAM17, future therapeutic strategies may benefit from temporally restricted, endothelial-targeted, locally delivered, or substrate-selective inhibition to minimize systemic side effects while preserving physiological ADAM17 functions [44].

Together, the convergence of endothelial-specific genetic deletion and differential pharmacological inhibition strengthens the concept that ADAM17 represents a central endothelial regulator and a promising therapeutic target in acute lung injury.

## Conclusions

Collectively, our findings demonstrate that endothelial ADAM17 functions as a central inflammatory amplifier in acute lung injury by coordinating neutrophil trafficking, endothelial barrier disruption, and inflammatory amplification. Our study provides mechanistic insight into how endothelial ADAM17 drives pulmonary inflammation and vascular injury, thereby highlighting ADAM17 as a compelling therapeutic target in ARDS.

## Supplementary Information

Supplementary Material 1

## Data Availability

The datasets used and/or analysed during the current study are available from the corresponding author on reasonable request.

## Abbreviations

ADAM17: A Disintegrin and Metalloproteinase 17
ANOVA: Analysis of variance
ARDS: Acute respiratory distress syndrome
AT1R: Angiotensin II Type 1 Receptor
BAL: Bronchoalveolar lavage
BCA: Bicinchoninic acid
BSA: Bovine serum albumin
CD49d: Integrin alpha 4
CD62L: L-selectin
cDNA: Complementary deoxyribonucleic acid
CREB: CAMP Response Element-Binding Protein
CXCL2/3: CXC Chemokine Ligand 2/3
DAB: 3,3′-Diaminobenzidine
DAPI: 4′,6-Diamidino-2-phenylindole
ELA2: Elastase 2
FADD: Fas-Associated Death Domain Protein
IL-10: Interleukin-10
IL-6R: Interleukin-6 Receptor
iRhom2: Inactive Rhomboid Protein 2
iTAP: IRhom Tail-Associated Protein
JAK1: Janus Kinase 1
JAM-A: Junctional adhesion molecule A
JNK: C-Jun N-terminal Kinase
LPS: Lipopolysaccharide
MAPK1: Mitogen-Activated Protein Kinase 1 (ERK2)
MAPK3: Mitogen-Activated Protein Kinase 3 (ERK1)
MPO: Myeloperoxidase
mRNA: Messenger ribonucleic acid
NF-κB: Nuclear Factor kappa B
p38: P38 Mitogen-Activated Protein Kinase
PBS: Phosphate-buffered saline
PFA: Paraformaldehyde
PSGL-1: P-selectin glycoprotein ligand-1
STAT3: Signal Transducer and Activator of Transcription 3
TNFR1: Tumor Necrosis Factor Receptor 1
TNFR2: Tumor Necrosis Factor Receptor 2
TNF-α: Tumor Necrosis Factor alpha
TRADD: Tumor Necrosis Factor Receptor-Associated Death Domain
TRAF2: Tumor Necrosis Factor Receptor-Associated Factor 2
TRAM: TRIF-Related Adaptor Molecule
TRIF: TIR-Domain-Containing Adapter-Inducing Interferon-β
VE-cadherin: Vascular endothelial cadherin
vWF: Von Willebrand Factor
