# Supplementary material for: Endothelial ADAM17 facilitates neutrophil migration and pulmonary microvascular permeability in acute lung inflammation
Source: J Biomed Sci. 2026 Sep 28;33:91. doi: 10.1186/s12929-026-01290-7 (PMC13617887; doi:10.1186/s12929-026-01290-7)
Supplement: Supplementary file 1 [file 12929_2026_1290_MOESM1_ESM.pdf]

Endothelial ADAM17 Facilitates Neutrophil Migration and Pulmonary Microvascular Permeability in Acute Lung Inflammation

- Supplemental data -

Anna Biedritzky[1], Carolin Kleinmaier[1], Anika Fuhr[1], Lisa Maria Herrmann[1], Kristian-Christos Ngamsri[1], Franziska Konrad[2], and Michael Koeppen[1]

[1] Department of Anesthesiology and Intensive Care Medicine, University Hospital of Tuebingen, Germany

[2] Center for Anesthesiology, Intensive Care Medicine, Perioperative Medicine and Pain Therapy Markgröningen, RKH regional clinics holding and services GmbH

Supplementary Figure 1

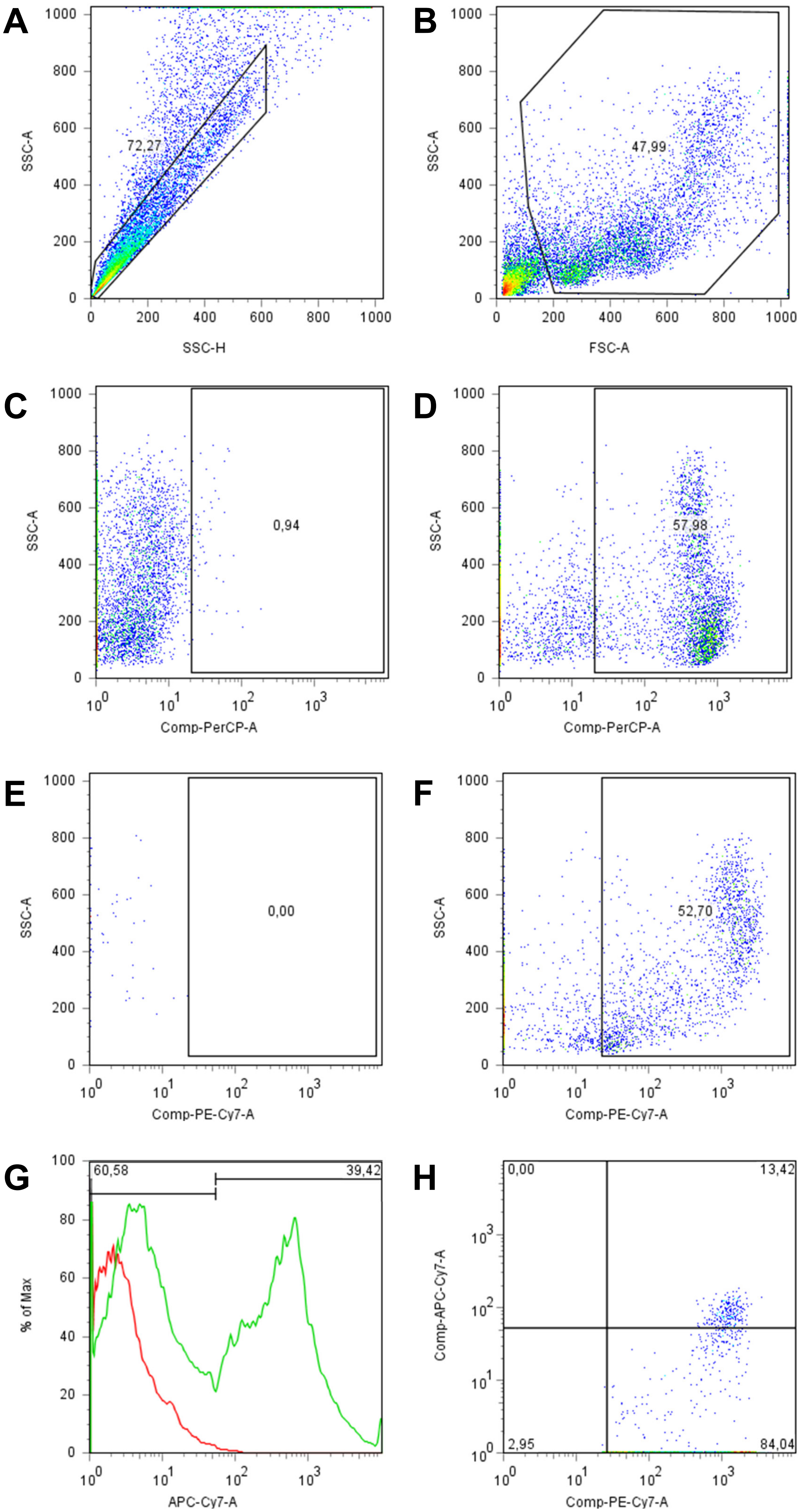

Supplementary Figure 1: Gating strategy for flow cytometry-based analysis of PMN distribution across the lung compartments using the example of a representative lung sample. **(A)** Single cell gating of all measured cells. **(B)** Gating of potential leukocytes based on size (FSC) and granularity (SSC) characteristics. **(C)** Gating of PerCP-CD45+ leukocytes based on the unstained sample. **(D)** Gating of PerCP-CD45+ leukocytes. **(E)** Gating of PE-Cy7-Ly6G+ PMNs based on the unstained sample. **(F)** Gating of PE-Cy7-Ly6G+ PMNs. The distinction between endothelial-adherent and interstitial PMNs followed. **(G)** For this purpose, the cut-off point between allophycocyanin/cyanine 7 $(APC\text{-}Cy7)^{neg}$ (red line) and $APC\text{-}Cy7^{pos}$ (green line) cells was set based on the compensation control of APC-Cy7. **(H)** Quadrant gate of PE-Cy7 and APC-Cy7 to identify PerCP-CD45+/PE-Cy7-Ly6G+/APC-Cy7-Ly6G+ endothelial-adherent PMNs (Q2) and PerCP-CD45+/PE-Cy7-Ly6G+/APC-Cy7-Ly6G- interstitial PMNs (Q4).

Supplementary Figure 2

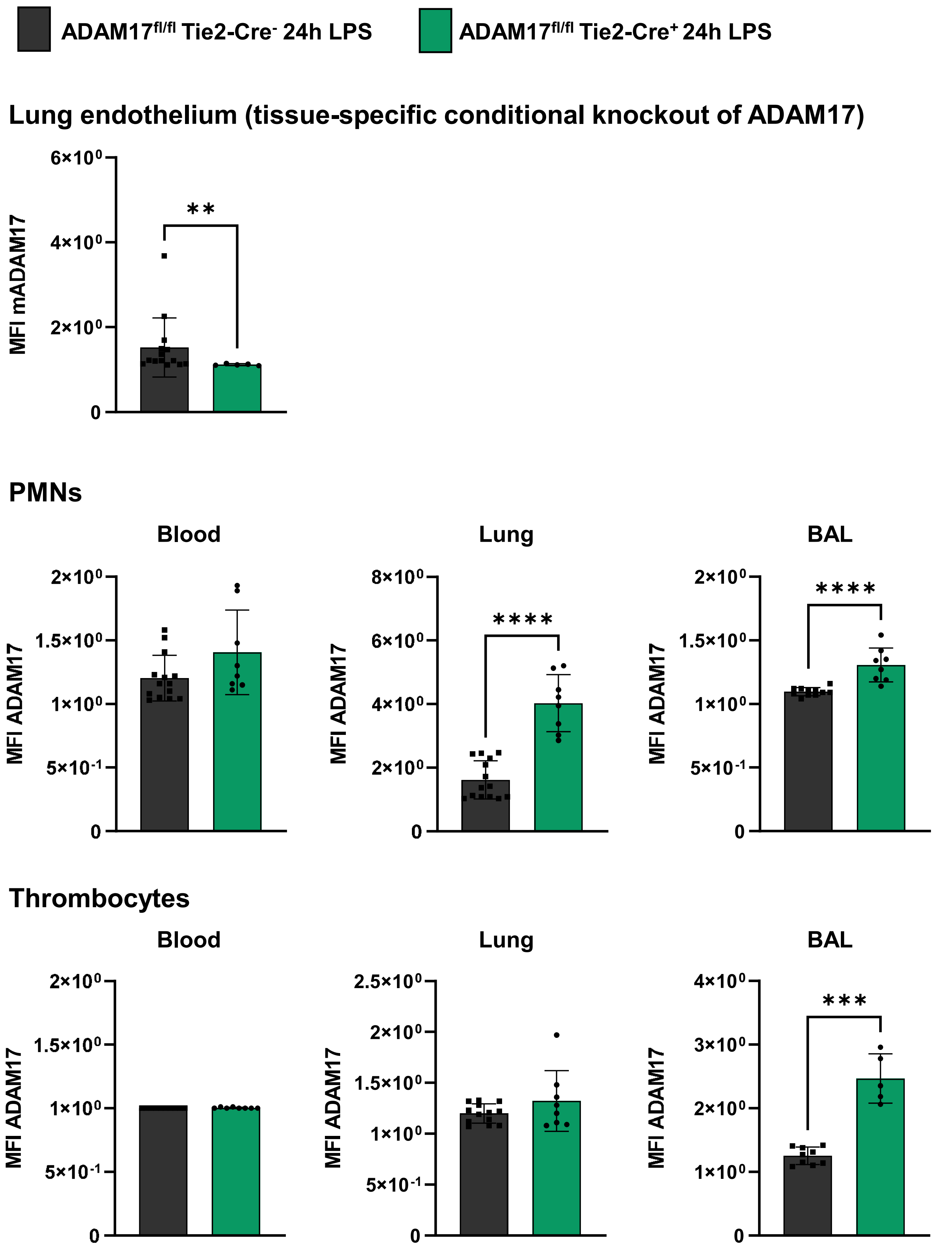

Supplementary Figure 2: Evidence of conditional knockout of A Disintegrin and Metalloproteinase 17 (ADAM17) on endothelial cells compared to PMNs and thrombocytes. **(A)** Flow cytometric quantification of the mean fluorescence intensity (MFI) of surface expression of ADAM17 on endothelial cells under the conditions mentioned. The MFI of ADAM17 on **(B)** PMNs and **(C)** thrombocytes was additionally analysed for comparison. (n=9-14/5-8). Data are presented as mean ± SD; Statistical analysis was performed using one-way ANOVA with appropriate post hoc testing, where appropriate, the Kruskal–Wallis test. *$P < 0.05$, **$P < 0.01$, ***$P < 0.001$, ****$P < 0.0001$.

Supplementary Figure 3

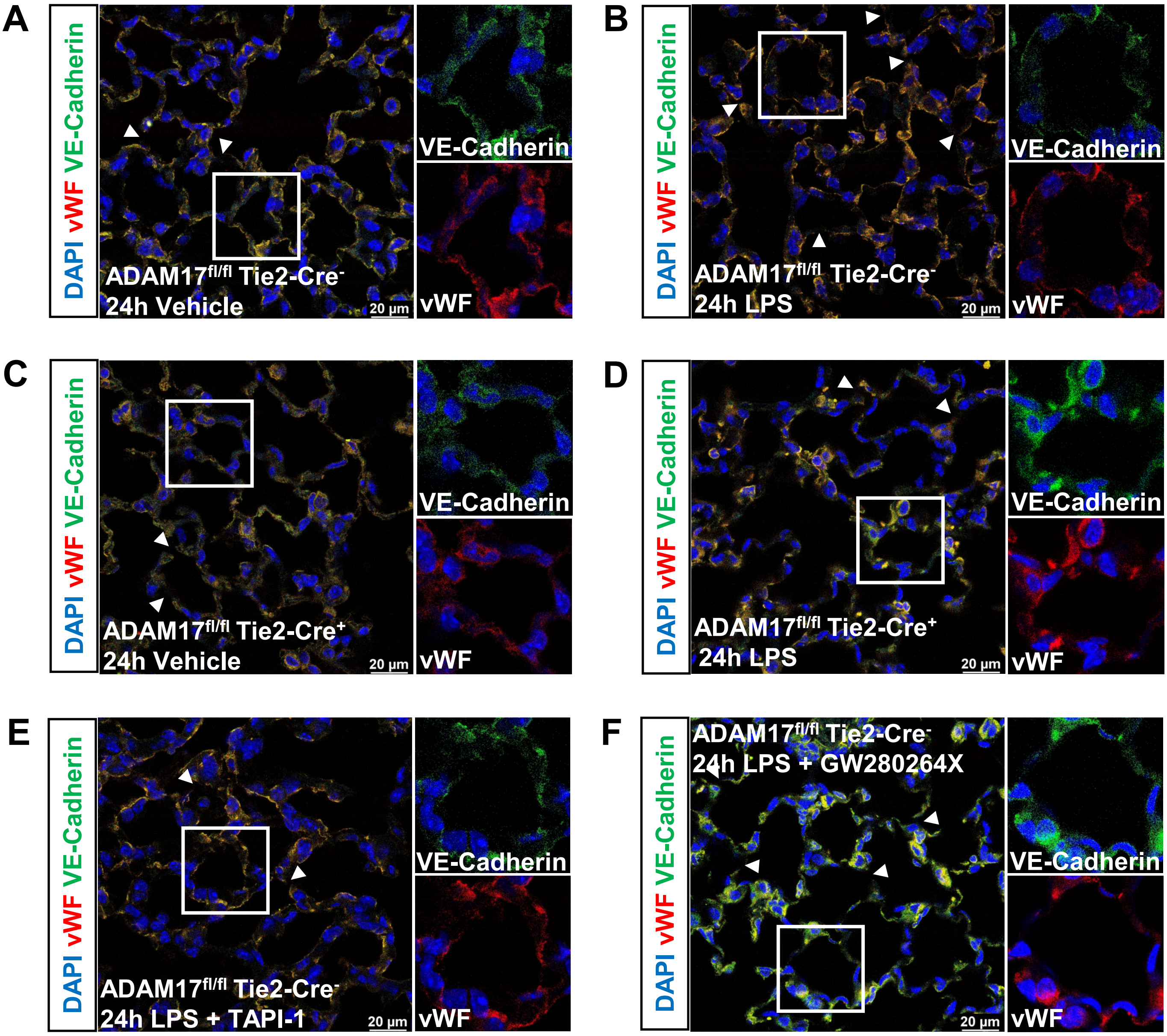

Supplementary Figure 3: (A-F) Representative immunofluorescence images showing VE-cadherin expression in murine lung tissue using LasX (n=4 mice per group; original magnification, x63). Pulmonary tissue of the indicated conditions was stained using an anti-VE-Cadherin antibody (green), with endothelial-specific anti-vWF staining (red) and nuclei staining (blue). Gaps between pulmonary endothelium were marked with with arrows.

Supplementary Figure 4

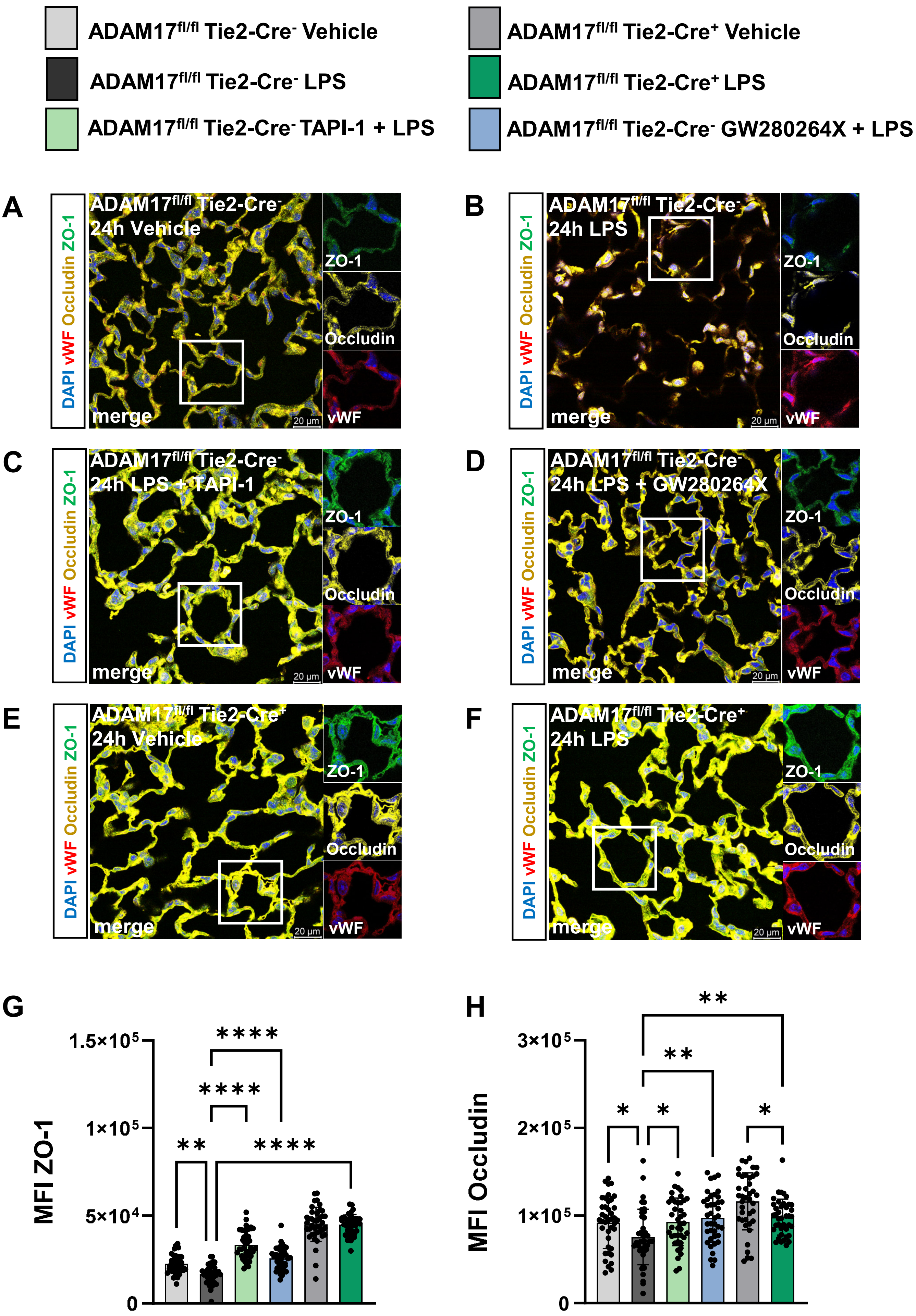

Supplementary Figure 4: (A-F) Representative immunofluorescence images of ZO-1 and occludin in murine lung tissue using LasX (n=4 mice per group; original magnification, x63). Pulmonary tissue of the indicated conditions was stained using an anti-ZO-1 (green) and anti-occludin antibody (yellow), with endothelial-specific anti-vWF staining (red) and nuclei staining (blue). Quantification of **(G)** ZO-1 and **(H)** occludin mean fluorescence intensity (MFI) in lung tissue using ImageJ software (n = 4 mice per group; 10 measurements per image). Data are presented as mean ± SD. Statistical analysis was performed using one-way ANOVA with appropriate post hoc testing, where appropriate, the Kruskal–Wallis test. $^{*}P < 0.05$, $^{**}P < 0.01$, $^{***}P < 0.001$, $^{****}P < 0.0001$.

Supplementary Figure 5

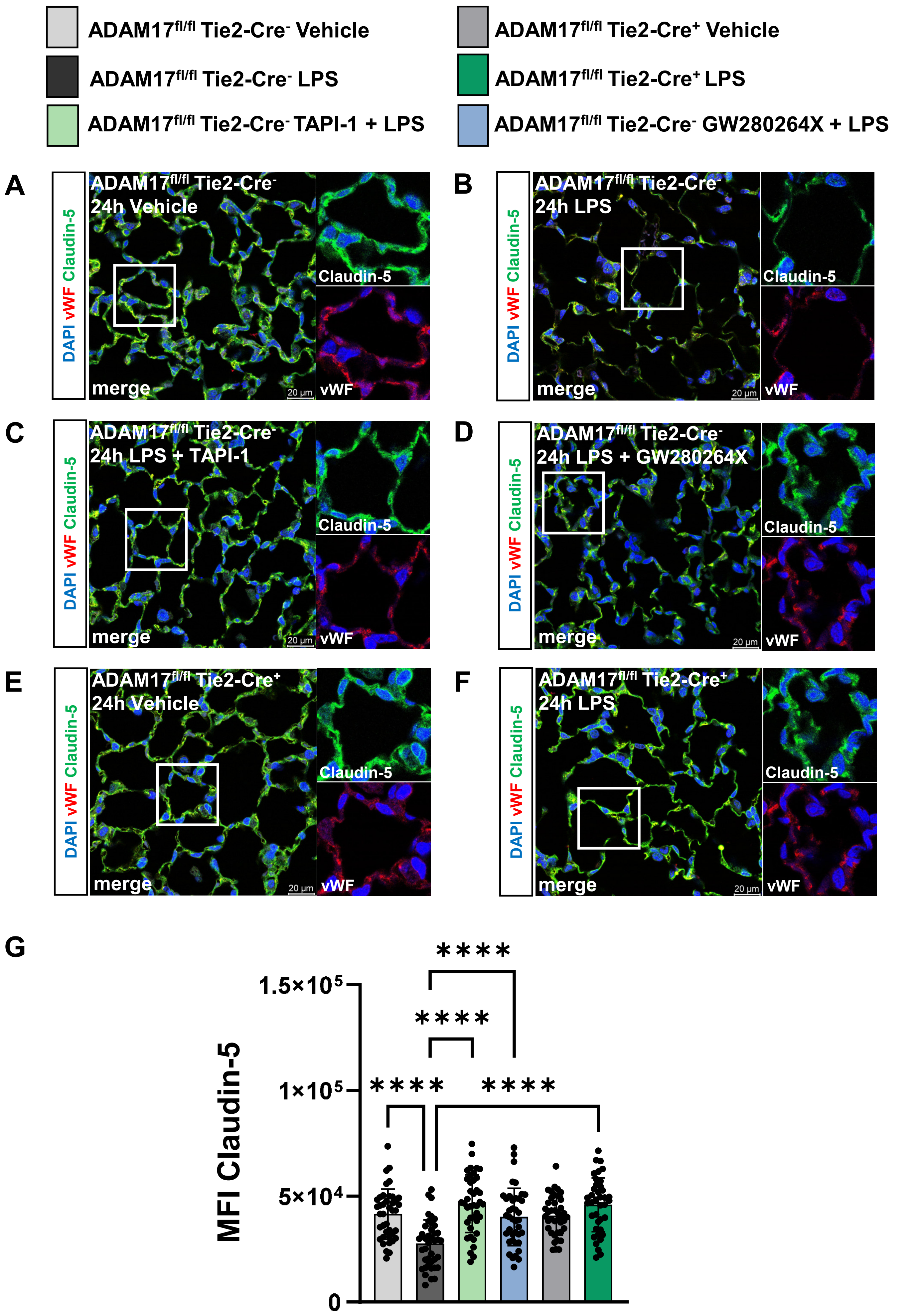

Supplementary Figure 5: (A-F) Representative immunofluorescence images of claudin-5 in murine lung tissue using LasX (n=4 mice per group; original magnification, x63). Pulmonary tissue of the indicated conditions was stained using an anti-claudin-5 (green) antibody, with endothelial-specific anti-vWF staining (red) and nuclei staining (blue). **(G)** Quantification of claudin-5 mean fluorescence intensity (MFI) in lung tissue using ImageJ software (n = 4 mice per group; 10 measurements per image). Data are presented as mean ± SD. Statistical analysis was performed using one-way ANOVA with appropriate post hoc testing, where appropriate, the Kruskal–Wallis test. ****P < 0.0001.

Supplementary Figure 6

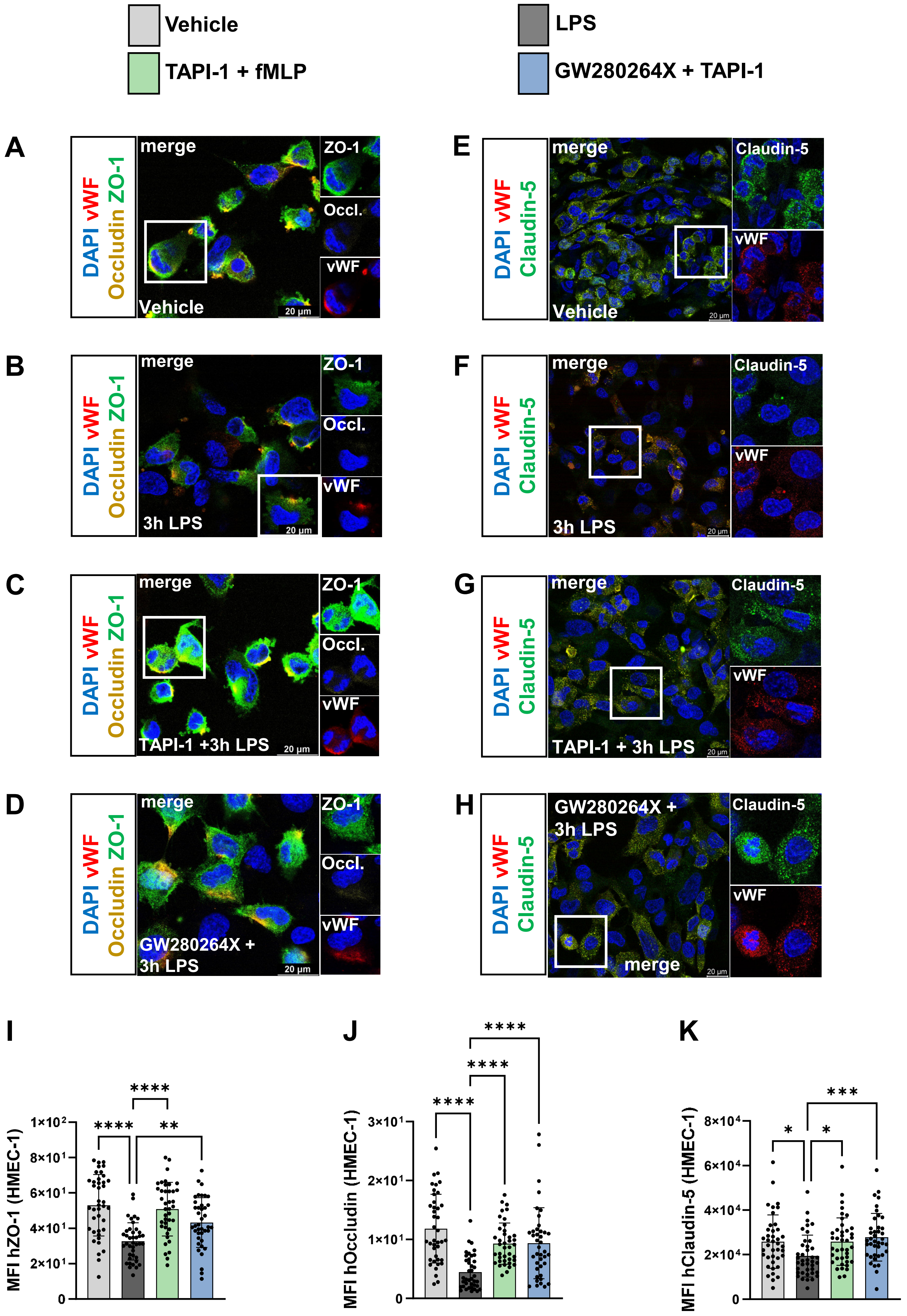

Supplementary Figure 6: Representative immunofluorescence images of **(A-D)** ZO-1, occludin and **(E-H)** claudin-5 in endothelial HMEC-1 cells using LasX (n=4 per group; original magnification, x63). HMEC-1 cells of the indicated conditions were stained using an anti-ZO-1 (green) and anti-occludin (yellow) as well as an anti-claudin-5 (green) antibody, with endothelial-specific anti-vWF staining (red) and nuclei staining (blue). Quantification of human **(I)** ZO-1, **(J)** occludin, and **(K)** claudin-5 mean fluorescence intensity (MFI) in HMEC-1 using ImageJ software (n = 4 per group; 10 measurements per image). Data are presented as mean ± SD. Statistical analysis was performed using one-way ANOVA with appropriate post hoc testing, where appropriate, the Kruskal–Wallis test. $^{*}P < 0.05$, $^{**}P < 0.01$, $^{***}P < 0.001$, $^{****}P < 0.0001$.

Supplementary Figure 7

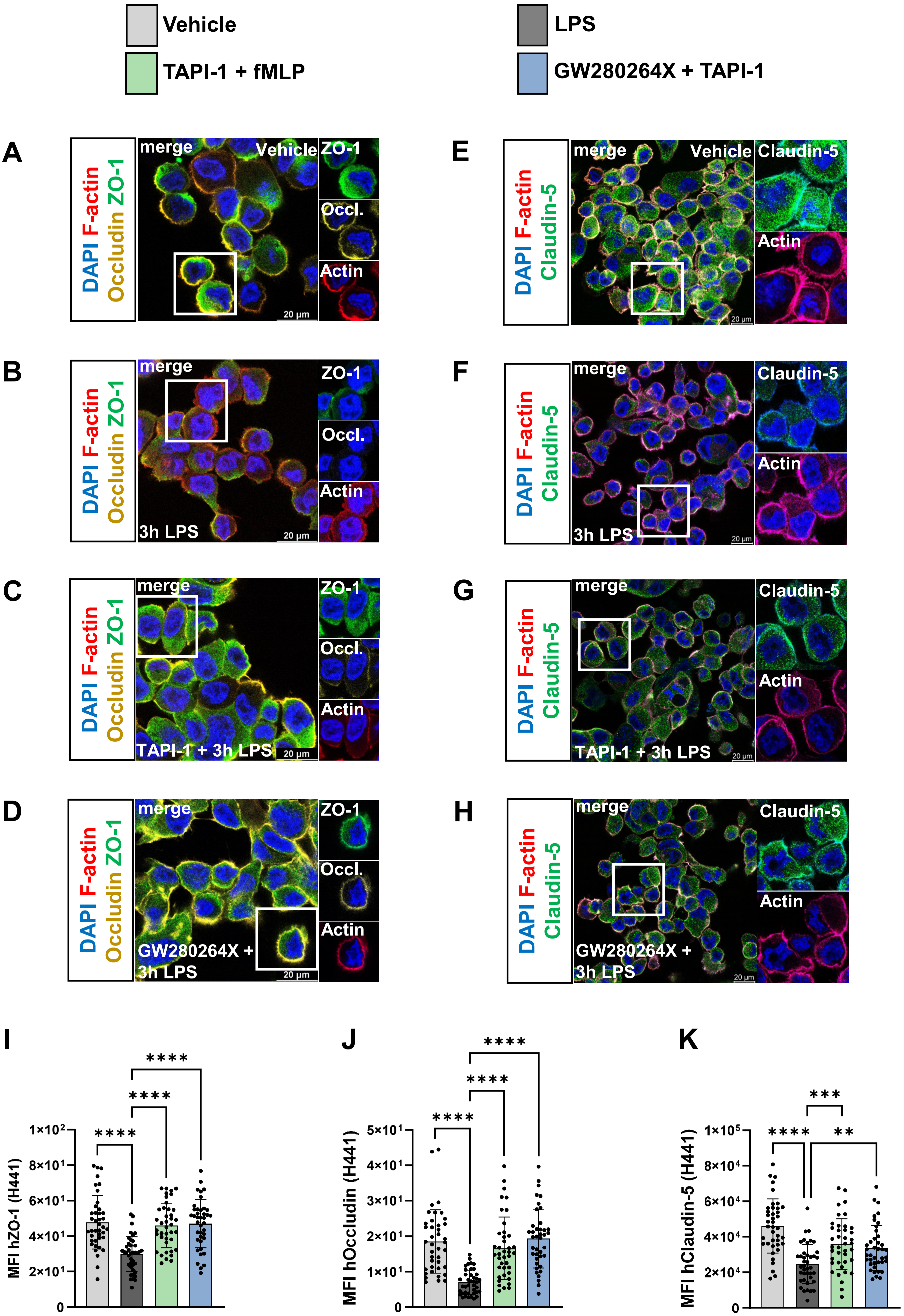

Supplementary Figure 7: Representative immunofluorescence images of **(A-D)** ZO-1, occludin and **(E-H)** claudin-5 in pulmonary epithelial H441 cells using LasX (n=4 per group; original magnification, x63). H441 cells of the indicated conditions were stained using an anti-ZO-1 (green) and anti-occludin (yellow) as well as an anti-claudin-5 (green) antibody, with epithelial-specific anti-F-actin staining (red) and nuclei staining (blue). Quantification of human **(I)** ZO-1, **(J)** occludin, and **(K)** claudin-5 mean fluorescence intensity (MFI) in H441 using ImageJ software (n = 4 per group; 10 measurements per image). Data are presented as mean ± SD. Statistical analysis was performed using one-way ANOVA with appropriate post hoc testing, where appropriate, the Kruskal–Wallis test. $^{**}P < 0.01$, $^{***}P < 0.001$, $^{****}P < 0.0001$.

Supplementary Figure 8

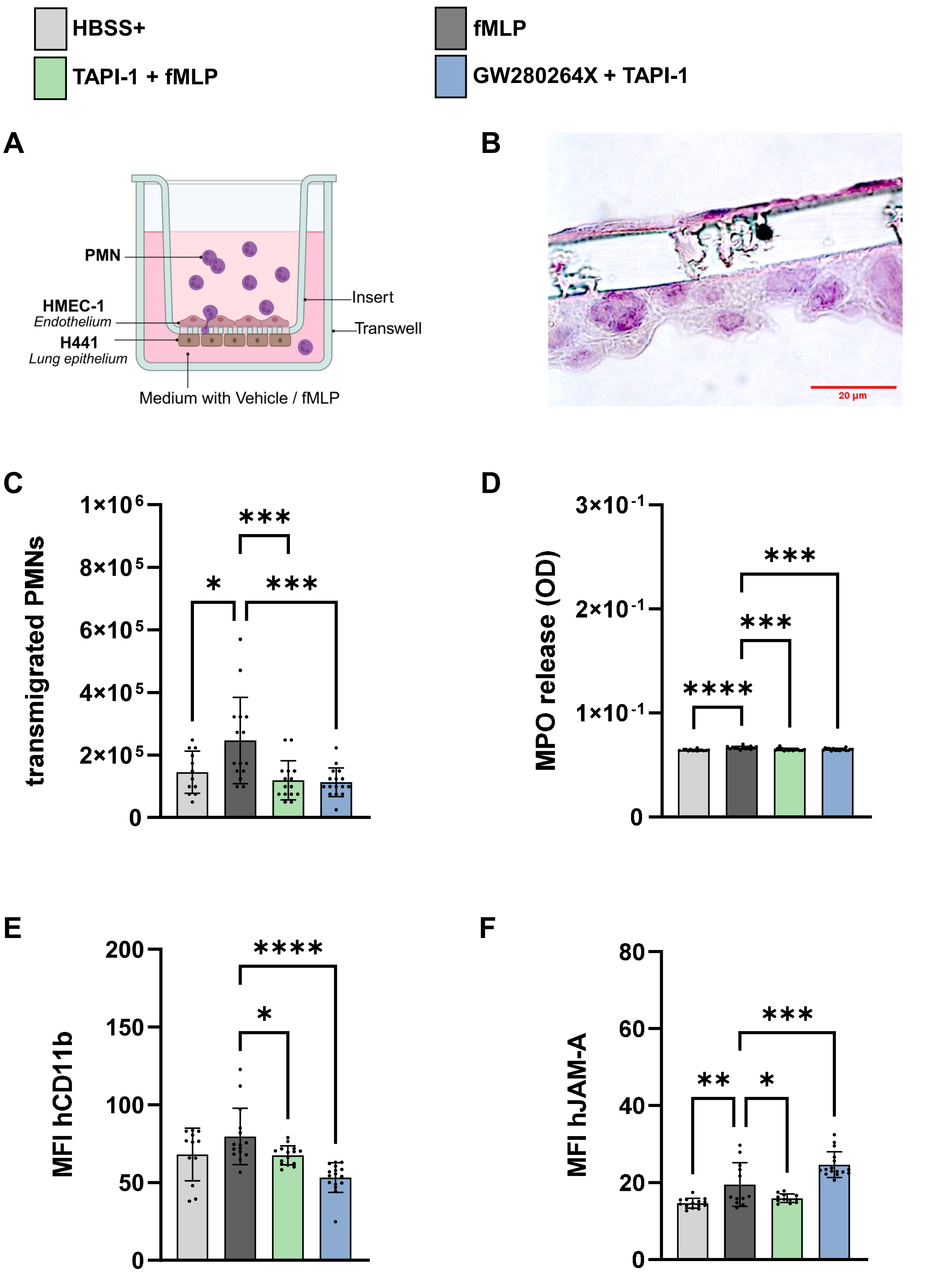

Supplementary Figure 8: (A) Schematic illustration of the in vitro transendothelial-transepithelial transmigration assay. HMEC-1 endothelial cells and H441 epithelial cells were cultured on opposite sides of Transwell inserts. Freshly isolated human neutrophils were added to the upper chamber and allowed to migrate toward an fMLP gradient (10 ng/mL). Prior to the assay, cell monolayers were pretreated with vehicle, the ADAM17-inhibitor TAPI-1, or the combined ADAM10/17-inhibitor GW280264X. **(B)** Representative histological section of a Transwell insert showing the endothelial and epithelial monolayers after completion of the transmigration assay. **(C)** Quantification of transmigrated neutrophils. **(D)** Myeloperoxidase (MPO) release into the supernatant. **(E–F)** Flow cytometric analysis of transmigrated neutrophils demonstrated reduced surface expression of **(E)** CD11b and **(F)** JAM-A following ADAM17 inhibition. Data are presented as mean ± SD (n=12-16). Statistical analysis was performed using one-way ANOVA with appropriate post hoc testing, where appropriate, the Kruskal–Wallis test. *P < 0.05, **P < 0.01, ***P < 0.001, ****P < 0.0001.

Supplementary Figure 9

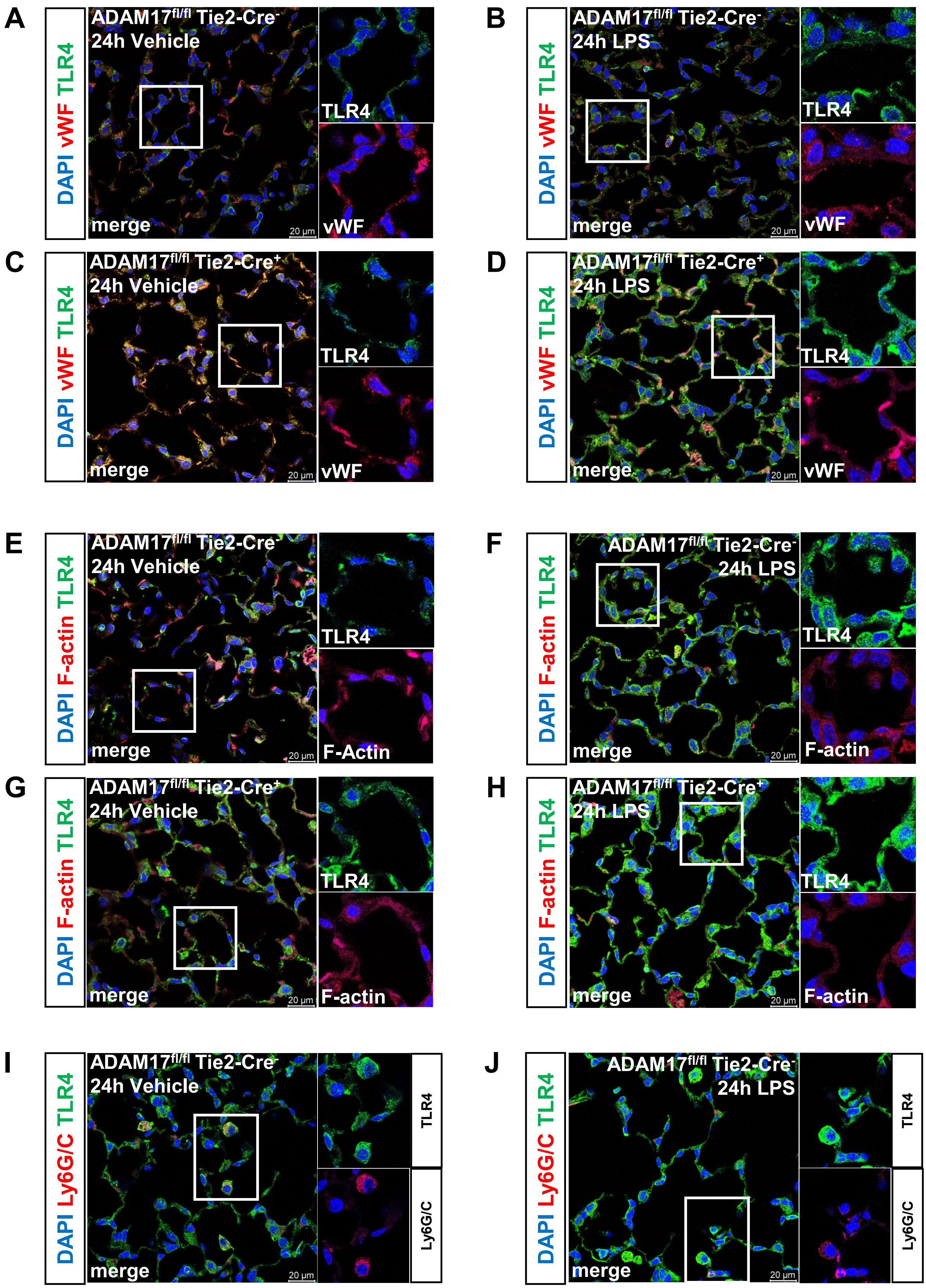

Supplementary Figure 9: Representative immunofluorescence images of TLR4 in murine lung tissue on **(A-D)** endothelial cells, on **(E-H)** epithelial cells and **(I-J)** infiltrating neutrophils within the alveolar space using LasX (n=3 per group; original magnification, x63). Tissue sections of the indicated conditions were stained using an anti-TLR4 (green) antibody with endothelial-specific anti-vWF staining (red), epithelial-specific anti-F-actin staining (red), neutrophil-specific anti-Ly6G/C-staining (red) and nuclei staining (blue).

Supplementary Figure 10

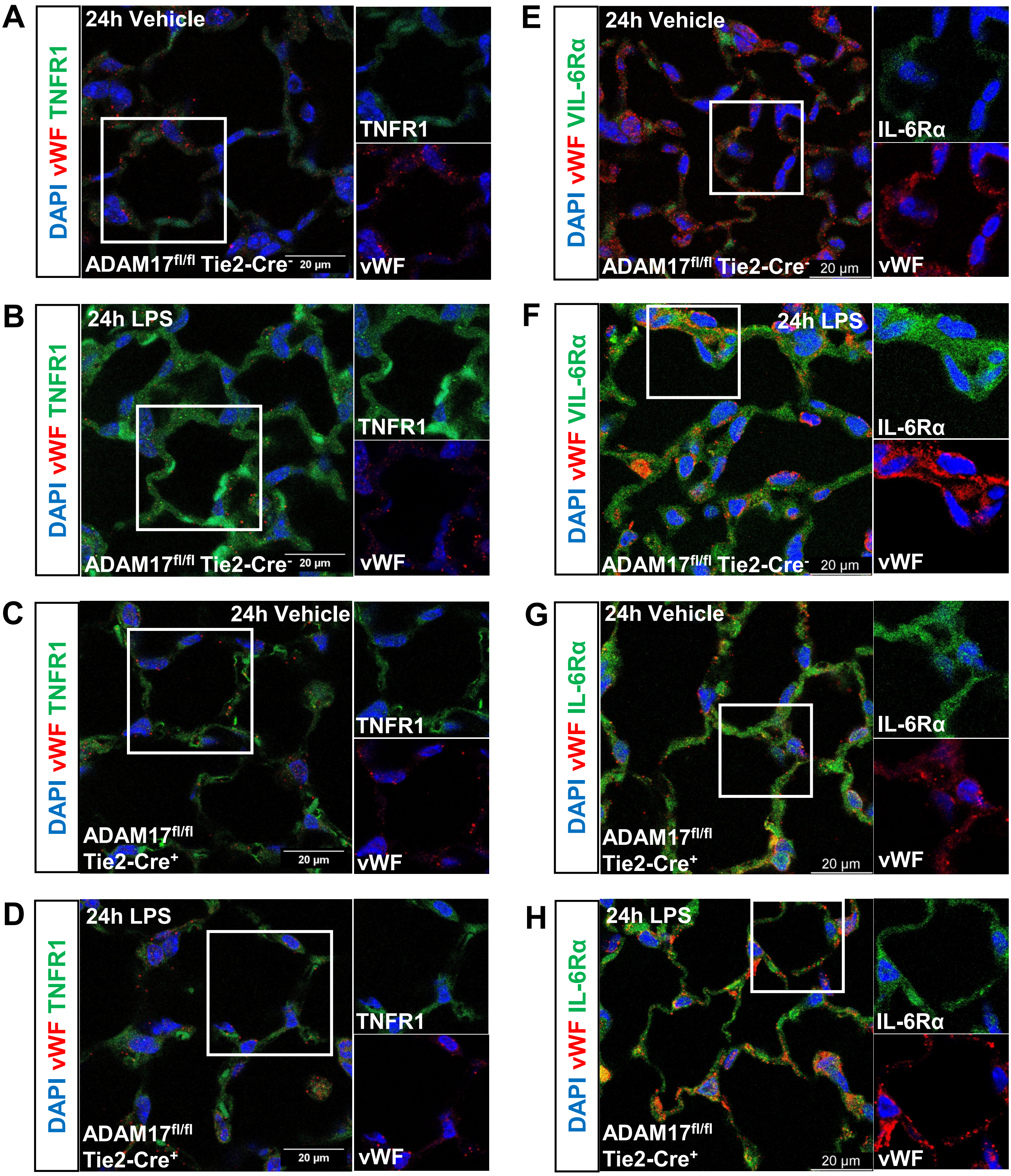

Supplementary Figure 10: Representative immunofluorescence images of **(A-D)** TNFR1 and **(E-F)** IL-6Rα expression in murine lung tissue using LasX (n=4 mice per group; original magnification, x63). Pulmonary tissue of the indicated conditions was stained using an anti-TNFR1 or anti-IL-6Rα antibody (green), with endothelial-specific anti-vWF staining (red) and nuclei staining (blue).
